# Human *ACVR1C* missense variants that correlate with altered body fat distribution produce metabolic alterations of graded severity in knock-in mutant mice

**DOI:** 10.1016/j.molmet.2024.101890

**Published:** 2024-02-01

**Authors:** Pawanrat Tangseefa, Hong Jin, Houyu Zhang, Meng Xie, Carlos F. Ibáñez

**Affiliations:** 1 Chinese Institute for Brain Research, Zhongguancun Life Science Park, 102206 Beijing, China; 2 Peking University School of Life Sciences, Peking-Tsinghua Center for Life Sciences, 100871 Beijing, China; 3 PKU-IDG/McGovern Institute for Brain Research, Beijing, China; 4 Peking University School of Psychological and Cognitive Sciences, 100871 Beijing, China; 5 Department of Biosciences and Nutrition, Karolinska Institute, Huddinge 14157, Sweden; 6 Department of Neuroscience, Karolinska Institute, Stockholm 17177, Sweden; 7 Stellenbosch Institute for Advanced Study, Wallenberg Research Centre at Stellenbosch University, Stellenbosch 7600, South Africa

**Keywords:** Adipose tissue, Lipolysis, Obesity, Single-nucleotide variant

## Abstract

**Background & aims:**

Genome-wide studies have identified three missense variants in the human gene *ACVR1C*, encoding the TGF-β superfamily receptor ALK7, that correlate with altered waist-to-hip ratio adjusted for body mass index (WHR/BMI), a measure of body fat distribution.

**Methods:**

To move from correlation to causation and understand the effects of these variants on fat accumulation and adipose tissue function, we introduced each of the variants in the mouse *Acvr1c* locus and investigated metabolic phenotypes in comparison with a null mutation.

**Results:**

Mice carrying the I195T variant showed resistance to high fat diet (HFD)-induced obesity, increased catecholamine-induced adipose tissue lipolysis and impaired ALK7 signaling, phenocopying the null mutants. Mice with the I482V variant displayed an intermediate phenotype, with partial resistance to HFD-induced obesity, reduction in subcutaneous, but not visceral, fat mass, decreased systemic lipolysis and reduced ALK7 signaling. Surprisingly, mice carrying the N150H variant were metabolically indistinguishable from wild type under HFD, although ALK7 signaling was reduced at low ligand concentrations.

**Conclusion:**

Together, these results validate ALK7 as an attractive drug target in human obesity and suggest a lower threshold for ALK7 function in humans compared to mice.

## Highlights

• Mice carrying I195T variant from human ACVR1C gene showed resistance to high fat diet (HFD)-induced obesity.
• Mice with the I482V variant displayed an intermediate phenotype.
• Mice carrying the N150H variant were metabolically indistinguishable from wild type under HFD.

## 1 Introduction

Following its discovery during the late 1990's [[1], [2], [3]], the TGF-β superfamily receptor ALK7, encoded by the *Acvr1c* gene, has emerged as an important regulator of adipocyte function and fat accumulation, primarily, through studies in mutant mice [4,5]. Although ALK7 is highly conserved between rodents and humans, it has remained unclear whether it may fulfill similar functions in human adipose tissue. Recently, several missense, non-coding and nonsense variants were identified in the human *ACVR1C* gene in independent sets of exome sequence data by different consortium studies [[6], [7], [8]]. An exome-wide association study of coding variants in over 400,000 individuals identified 4 variants in *ACVR1C* which correlated with changes in waist-to-hip ratio adjusted for body mass index (WHR/BMI), a measure of body fat distribution in humans [6]. Three of these were missense mutations that resulted in changes in the ALK7 protein sequence, namely N150H (rs55920843), I195T (rs56188432) and I482V (rs7594480). The variants were relatively rare, with minor allele frequencies (MAF) between 0.2 % and 7.2 %, and they all correlated with reduced WHR/BMI (P values between 10^−5^ and 10^−17^). Another study analyzed the association between WHR/BMI and predicted coding and splice site variants in over 340,000 individuals and found 9 rare variants (MAF<5 %) in different genes, among which was the N150H *ACVR1C* variant (MAF 1.1 %) [7]. A third study tested the cumulative burden of rare gene variants affecting fat distribution in 184,246 individuals from the UK Biobank and identified variants in 4 genes associated with beneficial effects on WHR/BMI among which was the I195T *ACVR1C* variant [8]. Whether and how these variants affect ALK7 function in adipocytes has not been investigated.

ALK7 plays fundamental roles in adipose tissue homeostasis [9]. Mice engineered with a constitutive null mutation in the *Acvr1c* locus show reduced fat accumulation and are resistant to diet-induced obesity [10]. Intriguingly, BALB/c mice lack a functional ALK7 protein and show resistance to diet-induced obesity compared to other strains [11]. Specific ablation of ALK7 in adipose tissue phenocopies these effects [4]. ALK7 signaling in adipocytes suppresses expression of adrenergic receptors thereby reducing catecholamine-induced lipolysis and enhancing fat accumulation [4]. This finding provided a long-sought link between nutrient overload and catecholamine resistance in obesity. Although elimination of ALK7 in already obese mice showed little effect on weight gain and fat accumulation, it synergized strongly with life-style change and anti-inflammatory interventions, enhancing lipolysis and energy expenditure and reducing adipose tissue mass and body weight gain, even under sustained high caloric intake [5]. This suggests that inhibition of ALK7 can be combined with simple interventions to produce longer-lasting benefits in obesity.

ALK7 is specifically expressed in mature adipocytes of both white and brown adipose tissue depots, with negligible levels in precursors and immature cells [12]. It signals in response to several members of the activin family, including activin B, C and E [[13], [14], [15], [16], [17]] as well as growth and differentiation factor 3 (GDF-3) [9]. ALK7 ligands may be produced in adipose tissue but also available to adipocytes from the blood stream. The levels of activin B and GDF-3 are increased in mice fed on a high fat diet (HFD) [[18], [19], [20]]. The main mediators of ALK7 intracellular signaling are Smad2 and Smad3 proteins, which upon phosphorylation by the ALK7 kinase migrate to the nucleus and regulate gene transcription in association with nuclear co-factors [9]. Through their interaction with C/EBPβ and C/EBPδ, these Smad proteins can repress the expression of C/EBPα, a master regulator of adrenergic receptor Adrb3 expression and catecholamine-induced lipolysis [21,22]. ALK7 intracellular signaling is likely to be more complex, including crosstalk to critical factors regulating, fasting, adipogenesis and adipocyte differentiation, such as PPARγ and PGC-1α [9,23].

Because the three missense variants identified in human *ACVR1C* correlated with reduced fat deposition, and previous mouse studies have shown that ALK7 is a positive regulator of fat accumulation, it could be speculated that the variants somehow negatively affect ALK7 expression or function. In the present study, we set out to investigate this by generating knock-in mice carrying each of the three variants and characterizing their metabolic phenotypes under both normal and high caloric diet regimes.

## 2 Materials and methods

### 2.1 Knock-in mice generation and maintenance

All mice were bred and group-housed (maximum 5 mice/cage) in pathogen-free conditions at the Chinese Institute for Brain Research (Beijing, China) under a 12 h light–dark cycle (lights on at 06:00) and constant temperature (20–23 °C) and ad libitum access to a standard chow diet and water. For diet-induced obesity studies, mice were fed a high fat diet (Research Diets #D12492: 60 % kcal from fat; NJ, USA) from 8 to 20 weeks of age. *Alk7*^fx/fx^ mice [4] were crossed to the *EIIa-cre* mice to generate the knock-out allele of *Acvr1c* used in the present studies. Animals were weighed twice weekly for the duration of the study and at the end of the study. Mice carrying I195T, I482V and N150H gene variants were generated by Cyagen Biosciences Inc, California, USA on a C57BL/6J background. Only male mice were used in this study. All studies were performed with Institutional Ethics approval.

### 2.2 Glucose and insulin tolerance tests

Glucose tolerance tests (GTTs) were performed following intraperitoneal injection of 1.5 g/kg glucose after overnight fasting. Blood glucose levels were measured at indicated time points using a handheld glucometer (Accu-Chek, Roche, China). For insulin tolerance tests (ITTs), mice were fasted for 6 h followed by intraperitoneal injection of 1U/kg insulin (Humulin R, Lilly). Glucose levels were determined at the indicated time points as above.

### 2.3 Whole-body energy homeostasis

Indirect calorimetry and food intake were determined using metabolic cues from Columbus Instruments (Columbus, OH, USA) as previously described (Guo 2014). Mice were housed individually with ad libitum access to a high fat diet and water. Mice were acclimatized to the metabolic cages for 24h prior to a 2–4 day period of automated data collection.

### 2.4 *In vivo* and *ex vivo* lipolysis

For *in vivo* lipolysis, mouse tail blood was taken from non-fasted mice for measurement of serum glycerol levels before and after intraperitoneal injection of the β3-AR-specific agonist CL-316,243 (ab144605, Abcam) (1 mg/kg body weight) at 20- or 40-minutes. For *ex vivo* lipolysis, epididymal or inguinal adipose tissue pieces weighing about 50 mg were isolated from overnight fasted mice. Explants were washed twice in PBS and incubated in Krebs–Ringer Bicarbonate Buffer (KRBH) containing 1 % fatty acid-free BSA (Sigma-Aldrich). The samples were treated with either vehicle or CL-316,243 (100 μM) for 2 h at 37 °C with mild shaking at 150rpm. After incubation, glycerol release was measured using a free glycerol reagent (Sigma-Aldrich).

### 2.5 Tissue isolation, adipocyte differentiation and treatment

Stromal-vascular fractions (SVF) were isolated as previously described [5,12]. Briefly, inguinal white adipose tissues were dissected from 8-week-old mice, minced and digested in 1.5 %BSA KRBH containing 1 mg/ml collagenase I (Solarbio, China) at 37 °C for 1h. Digested tissue was filtered through 100 μm nylon mesh and centrifuged to separate floating adipocytes. The resulting pellet (SVF) was cultured in DMEM, supplemented with 10 % FBS, 1 % MEM non-essential Amino acid solution (11140050, Gibco), 1 % GlutaMax (35050061, Gibco) and 100U/ml penicillin and streptomycin (growth medium). Adipogenesis was induced by incubation in growth medium supplemented with 1x ITS (Gibco), 1 μM dexamethasone, 33 μM Biotin, 2 nM T3, 17 μM Pantothenate, 0.5 mM IBMX and 1 μM rosiglitazone (differentiation medium). After 2–4 days, the medium was changed to differentiation medium without IBMX and rosiglitazone (maintenance medium), and then changed again every 2 days until full differentiation (i.e., day 8–10). For activin B treatments, matured adipocytes were serum starved for 2h and then incubated with indicated concentration of activin B for either 20min for detection of SMAD phosphorylation levels, or 24h for RNA extraction. For CL-316,243 treatment, matured adipocytes were incubated with 10 μM CL-316,243 for 20min before harvested for protein extraction.

### 2.6 RNA isolation and quantitative RT-PCR

All RNA extractions were carried out using TRIzol reagent (Sigma) according to manufacturer's instructions. Total RNA (1.5 μg) was reverse transcribed into cDNA using Maxima H Minus First Strand cDNA Synthesis Kit (ThermoFisher, China). Real-time PCR reactions were performed using PowerUp™ SYBR™ Green reagent (Applied Biosystems, China) in a CFX Connect™ Real-Time PCR machine (Bio-Rad). Forward and reverse primer pairs are listed in Supplementary Table 1. Relative mRNA expression was determined using the 2–ΔΔCt method [32].

### 2.7 Leptin ELISA

Blood samples were collected (between 12 and 2PM) via cardiac puncture and centrifuged at 845×*g* for 10 min at room temperature and extracted serum stored at −80 °C. Commercial ELISA kits were used for the measurement of leptin (CSB-E04650m, Cusabio, China) as per manufacturer's instructions.

### 2.8 Histology

Tissues were dissected, fixed in 10 % formalin and embedded in paraffin. Hematoxylin and eosin (H&E) staining was performed on 5 μm adipose tissue or liver sections. Image J was used to measure adipocyte cell size and the distribution of adipocyte size was presented as a percentage of total cells from at least n = 5 images per mouse.

### 2.9 Protein isolation, immunoprecipitation and Western blotting

Snap-frozen adipose tissue or cell samples were homogenized in RIPA buffer (R0278, Sigma, China) with addition of protease inhibitors (Complete™, Roche, Basel, Switzerland) and centrifuged at 15000×*g* for 15 min to collect supernatants. Mature adipocytes were isolated from dissociated inguinal WAT by differential centrifugation. Equal amounts of protein (30 μg) were resolved by SDS-PAGE and transferred to Immun-Blot PVDF Membrane (BioRad). Mouse monoclonal antibody #180F7 against ALK7 extracellular domain was made in-house. Other antibodies (all from Cell Signaling Technology unless indicated otherwise) are phospho- Smad2/3 (#8828), total Smad2/3 (#3102), phospho-HSL (Ser^660^, #45804), total HSL (#4107), GAPDH (#G9545, Sigma) and hemagglutinin (HA) tag (#05-904, Sigma). Quantitative analysis was performed using ImageQuant software. Protein levels were quantified, and levels normalized to a loading control.

### 2.10 Cell transfection and immunoprecipitation

HEK293T cells were cultured in DMEM High Glucose (Gibco, Cat. No. C11995500BT) supplemented with 10 % FBS and 1 % Penicillin-Streptomycin. Transfection was conducted in 10 cm plates when cells reached 70 % confluence. 2 μg Flag-tagged Smad3 (OriGene, Cat. No. RR201118) and 8 μg of either wild type, I195T, N150H or I482V HA-tagged ALK7 expression plasmids were co-incubated with 40 μg polyethylenimine (PEI) in culture medium at room temperature for 10 min. Negative controls without either ALK7 or Smad3 were also included. The plasmid-PEI mixture was added to the cell monolayers and kept for 2 days. Cells were then washed once using ice-cold PBS and lysed by Pierce IP Lysis/Wash buffer (Thermo Scientific, Cat. No. 87787) on the ice for 5 min with periodic mixing. Cell lysates were centrifuged at 12,000 rpm at 4 °C for 10 min, and the concentration of protein samples was determined using a BCA protein assay kit (Solarbio, Cat. No. PC0020). For immunoprecipitations, 1 mg whole cell lysate was combined with 1 μg rabbit HA antibody (CST, Cat. No. 3724) at RT for 2 h with mixing, then 0.25 mg Pierce Protein A/G Magnetic Beads (Thermo, Cat. No. 88802) was used to bind the immune complex at RT for 1 h with mixing. After washing by IP Lysis/Wash buffer for three times, the HA-tagged protein was eluted from the beads by the Elution Buffer at RT with mixing for 10 min. For precipitation of His-tagged FKBP12, 1.5 mg protein lysate was incubated with 0.25 mg HisPur™ Ni-NTA Beads (Thermo Scientific, Cat. No. 88831) overnight at 4 °C with mixing. After washing with IP Lysis/Wash buffer for three times, the His-tagged protein was eluted from the beads by the Elution Buffer at RT with mixing for 10 min.

### 2.11 Statistical analysis

Statistics analyses were performed using Prism 9 and Graph Plot (GraphPad Software, LLC.). Data are expressed as average ± SEM unless otherwise indicated. Sample sizes were determined a priori as N = 3-4 for *in vitro* studies, N = 5-6 for histological studies, and N = 8-10 for whole animal studies based on previous work from our laboratory and other studies in the field. Group comparisons were made using one-way or two-way ANOVA as appropriate, followed by Tukey's post-hoc test. To account for differences in body weight between strains, ANCOVA was used for statistical analysis of indirect calorimetry measurements (https://www.mmpc.org/shared/regression.aspx). Differences were considered significant when p ≤ 0.05.

## 3 Results

### 3.1 Missense variants in human *ACVR1C* afford different levels of resistance to diet-induced obesity and fat accumulation in mice

**Figure 1:**
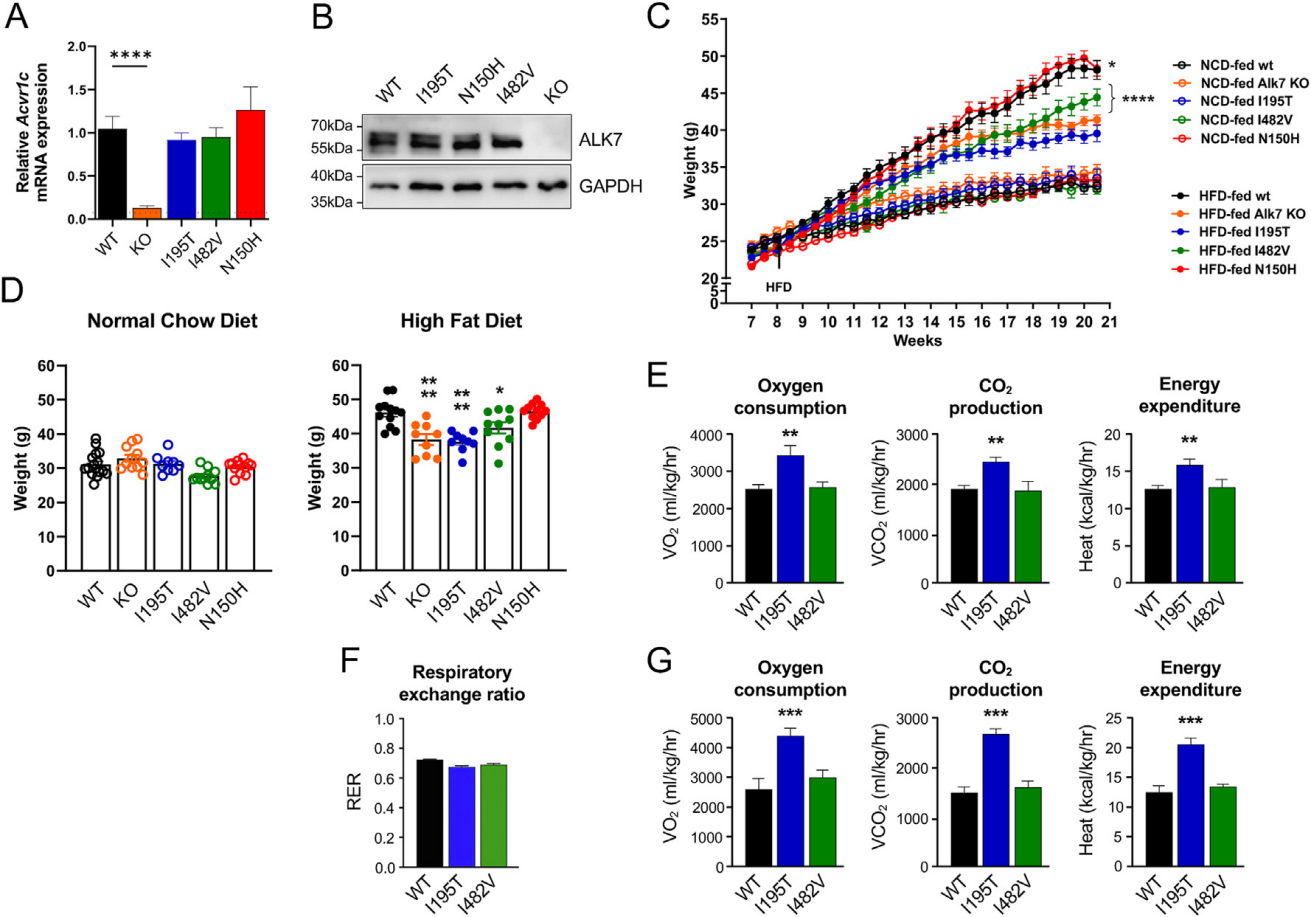
**Weight gain and energy consumption of knock-in mice carrying missense variants in *Acvr1c***. (A) mRNA levels assessed by quantitative RT-PCR for *Acvr1c*, encoding ALK7, in WAT of adult male mice. Data are presented as mean ± SEM of relative mRNA levels (normalized to wild type levels). ∗∗∗∗, p < 0.0001; N = 5 mice per genotype (one-way ANOVA with Tukey's post-hoc test). (B) Representative Western blot of ALK7 protein expression in mature adipocytes purified form inguinal WAT of wild type (WT), I195T, N150H, I482V and knock-out (KO) male mice. Blot with glyceraldehyde phosphate dehydrogenase (GAPDH) was used as loading control. (C) Weight gain of wild type mice (wt), Alk7 knock-out mice (KO) and mice carrying missense variants fed on a normal chow diet (NCD) or high-fat diet (HFD) from 8 weeks of age (arrow) and for an additional 12.5 weeks. Data are presented as mean ± SEM. ∗, p < 0.05; ∗∗∗∗, p < 0.0001 vs. wt at 20.5 weeks (end of study); N = 10 mice per genotype (two-way ANOVA with Tukey's post-hoc test). (D) Final body weight at 20.5 weeks of age. Data are presented as mean ± SEM. ∗, p < 0.05; ∗∗∗∗, p < 0.0001 vs. wild type (wt); N = 10 mice per genotype (one-way ANOVA with Tukey's post-hoc test). (E) Average oxygen consumption, CO2 production and energy expenditure in wild type (WT), I195T and I482V mice fed on HFD for 12.5 weeks. Data are presented as mean ± STDEV. N = 5 mice per genotype. ∗∗, p < 0.01 vs. wild type (ANOVA). (F) Respiratory exchange ratio in wild type (WT), I195T and I482V mice fed on HFD for 12.5 weeks. Data are presented as mean ± SEM. N = 5 mice per genotype. (G) Oxygen consumption, CO2 production and energy expenditure in wild type (WT), I195T and I482V mice after accounting for differences in body weight. Data are presented as average ± SEM. N = 5 mice per genotype. ∗∗∗, p < 0.001 vs. wild type (ANCOVA).

Three missense variants in human *ACVR1C* (N150H, I195T and I482V) were introduced as point mutations in the endogenous *Acvr1c* locus of C57Bl/6 mice (Supplementary Figs. S1A–C and Appendix). The sequences of human and mouse ALK7 proteins are nearly identical in the intracellular domains, including the positions affected by the three variants (Supplementary Fig. S2). Mice homozygous for the variants were viable and fertile. Analysis of mRNA expression in white adipose tissue (WAT) showed that all *Acvr1c* variants expressed at levels comparable to the wild type (Figure 1A). As expected, no *Acvr1c* mRNA could be detected in Alk7 KO adipose tissue. ALK7 protein was expressed at levels comparable to wild type in mature adipocytes from all three lines of mutant mice (Figure 1B). Body weight curves showed normal growth under chow diet for all three lines of mutant mice compared to wild type (Figure 1C,D). Mice carrying a null mutation in *Acvr1c* (Alk7 KO) also showed comparable body weight growth during the same period (Figure 1C,D). When placed on a high fat diet (HFD) at 8 weeks of age for 12 weeks, Alk7 KO mice gained significantly less weight than wild type mice (Figure 1C,D), in agreement with previous results [10]. I195T mice showed similar resistance to diet induced obesity as the null mutants, while I482V mice displayed an intermediate phenotype (Figure 1C,D). However, the growth rate and final body weight of N150H mice were indistinguishable from wild type mice under HFD (Figure 1C,D). I195T mice showed increased oxygen consumption, CO_2_ production and energy expenditure (Figure 1E–F), in agreement with previous observations in mice carrying a conditional *Acvr1c* null mutation in adipose tissue [4]. ANCOVA indicated that I195T mice have statistically significant higher energy expenditure than wild type even after accounting for their differences in body weight (Figure 1G). Energy expenditure in I482V mice was not different from wild type (Figure 1E–G). Food and water consumption was normal across these strains (not shown).

**Figure 2:**
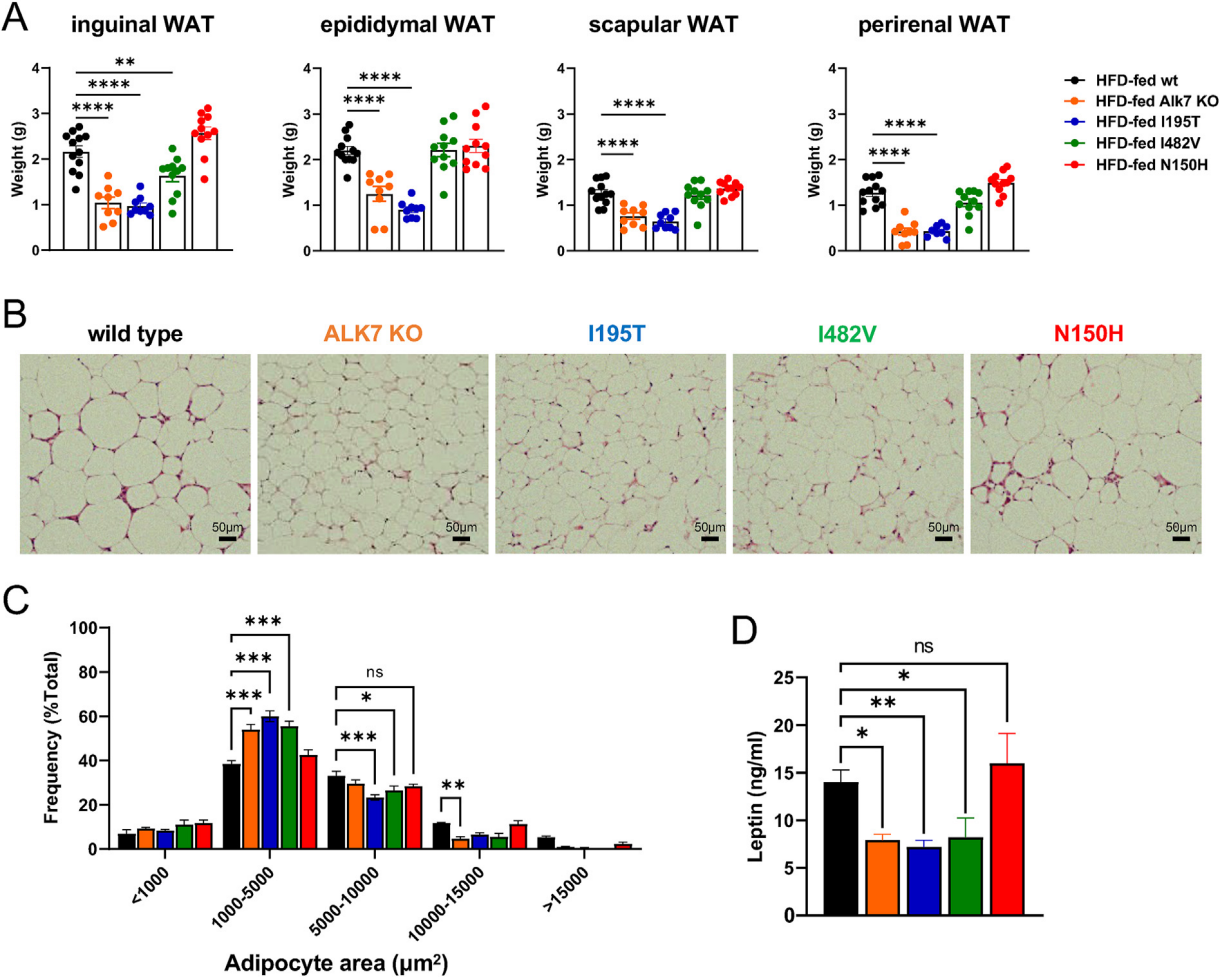
**Fat accumulation in wild type and ALK7 mutant mice after exposure to high-fat diet**. (A) Wet-weight of inguinal, epididymal, scapular and perirenal white adipose tissue (WAT) in wild type (wt), Alk7 knock-out (KO) and I195T, I482V and N150H mice after 12.5 week high fat diet (20.5 weeks of age). Data are presented as mean ± SEM. ∗∗, p < 0.01; ∗∗∗∗, p < 0.0001; N = 10 mice per genotype (one-way ANOVA with Tukey's post-hoc test). (B) Morphology of adipocytes in epididymal WAT of the indicated genotypes (H&E staining) after HFD. Scale bar, 50 μm. (C) Size distribution of adipocytes in epididymal WAT of the indicated genotypes after HFD. Data are presented as mean ± SEM. ns, not significant difference; ∗, p < 0.05; ∗∗, p < 0.01; ∗∗∗, p < 0.001; n = 5 sections per animal, N = 5 mice per genotype (two-way ANOVA with Tukey's post-hoc test). (D) Leptin levels in serum in the indicated genotypes after HFD. Data are presented as mean ± SEM. ns, not significant difference; ∗, p < 0.05; ∗∗, p < 0.01; N = 6 mice per genotype (one-way ANOVA with Tukey's post-hoc test).

Inguinal, epididymal, scapular and perirenal WAT depots were all significantly smaller in Alk7 KO and I195T mice after 12-week HFD compared to wild type mice (Figure 2A). In I482V mice, only the inguinal WAT depot was reduced compared to wild type, while N150H mice showed no difference in any of the depots compared to wild type mice (Figure 2A). In epididymal WAT, ALK7 KO, I195T and I482V mice showed altered size distribution of adipocytes, with a higher proportion of small and lower proportion of large adipocytes (Figure 2B,C), indicative of reduced lipid accumulation. Again, N150H mice were not different from wild type. In agreement with this, Alk7 KO, I195T and I482V mice, but not N150H, showed reduced levels of leptin in plasma (Figure 2D). Thus, I195T mice phenocopy Alk7 KO mice in their resistance to diet-induced obesity and fat accumulation, I482V mice displayed a milder phenotype, while N150H mice were indistinguishable from wild type mice.

### 3.2 Hyperinsulinemia, enhanced adipose tissue lipolysis and decreased adipose tissue inflammation in knock-in mice carryingt human *ACVR1C* variants

**Figure 3:**
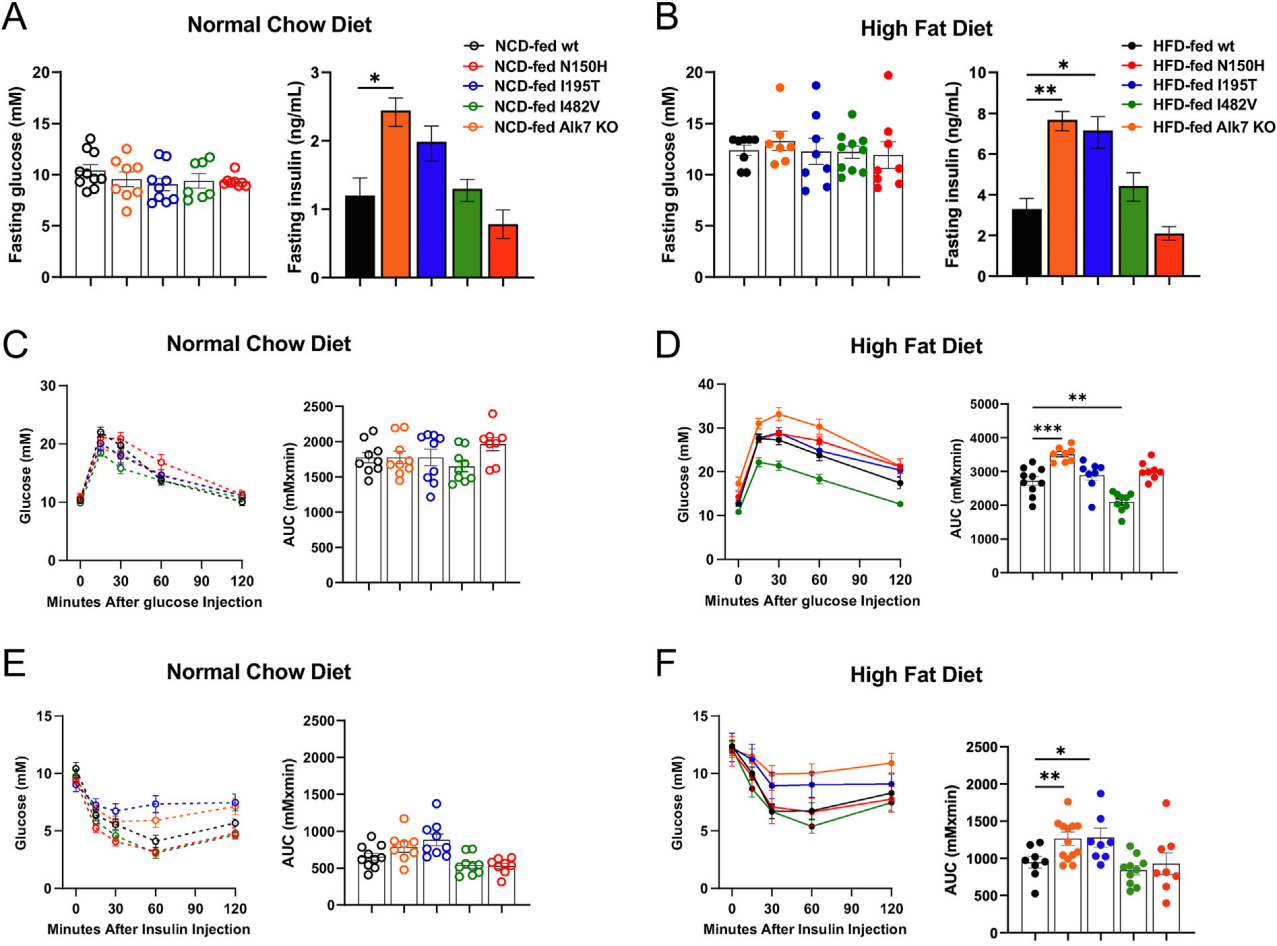
**Glucose and insulin responses in wild type and ALK7 mutant mice**. (A, B) Plasma glucose levels after 6 h fasting in 20.5 week old wild type and ALK7 mutant mice kept in normal chow diet (A) or after 12 week in HFD (B). Data are presented as mean ± SEM. ∗, p < 0.05; N = 6 mice per genotype (one-way ANOVA with Tukey's post-hoc test). (C, D) Glucose tolerance test in 20.5 week old wild type and ALK7 mutant mice kept in normal chow diet (A) or after 12 week in HFD (B). Data are presented as mean ± SEM. AUC, area under the curve. ∗∗, p < 0.01; ∗∗∗, p < 0.001; N = 10 mice per genotype (one-way ANOVA with Tukey's post-hoc test). (E, F) Insulin tolerance test in 20.5 week old wild type and ALK7 mutant mice kept in normal chow diet (A) or after 12 week in HFD (B). Data are presented as mean ± SEM. AUC, area under the curve. ∗, p < 0.05; ∗∗, p < 0.01; N = 10 mice per genotype (one-way ANOVA with Tukey's post-hoc test).

**Figure 4:**
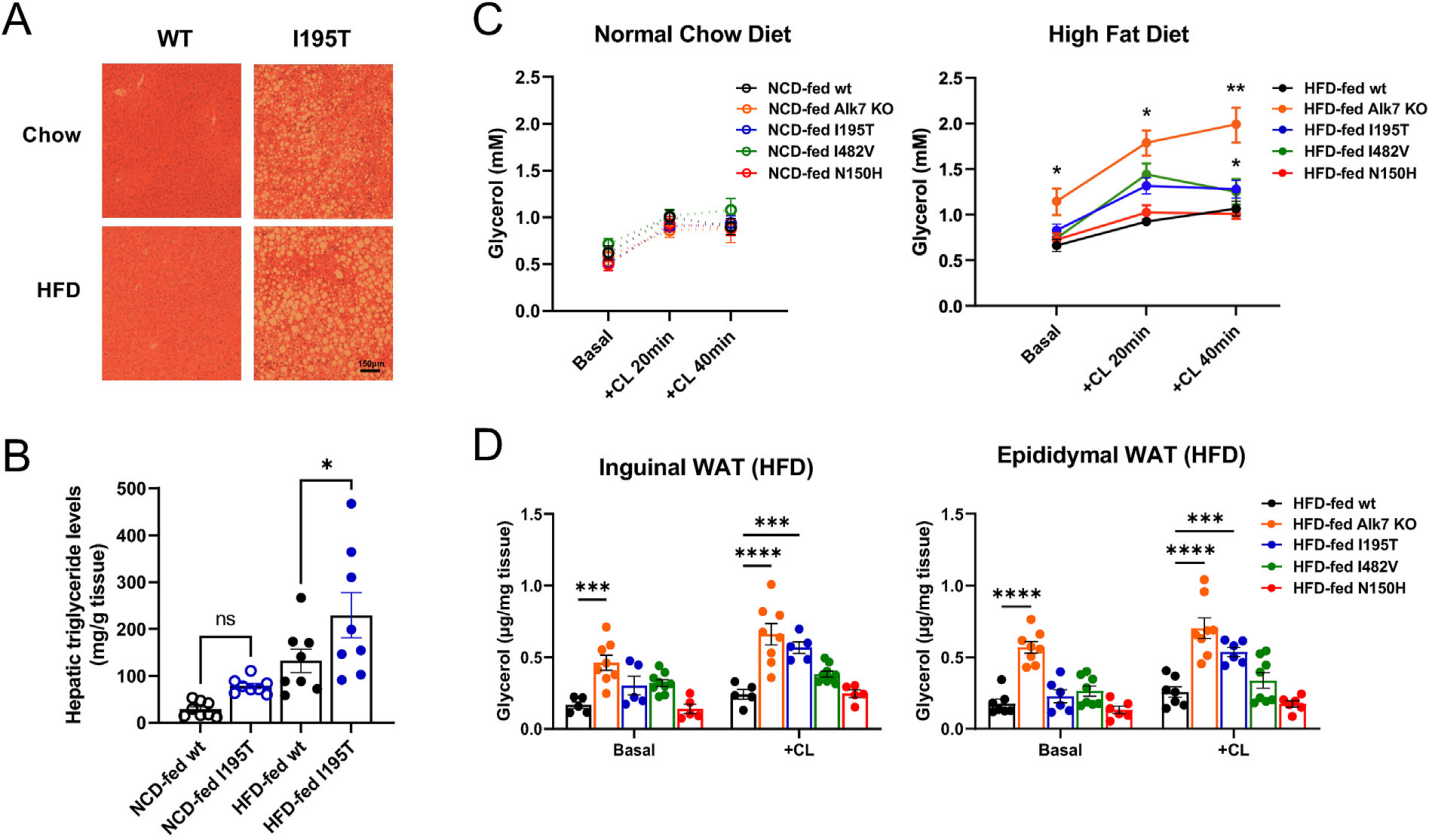
**Liver fat accumulation and adipose tissue lipolysis in wild type and ALK7 mutant mice**. (A) Representative images of H&E staining of liver sections of wild type and I195T mice after chow or high-fat diet (HFD). Scale bar, 150 μm. (B) Triglyceride levels in liver of wild type and I195T mice after chow (left) or HFD (right). Data are presented as mean ± SEM. ns, not signifiant difference; ∗, p < 0.05; N = 8 mice per genotype (one-way ANOVA with Tukey's post-hoc test). (C) *In vivo* lipolysis induced by i.p. injection of agonist to adrenergic receptor Adrb3 CL-316,243 (CL) in 20.5 week old wild type (wt) and ALK7 mutant mice kept in normal chow diet or after 12 week in HFD. Data are presented as mean ± SEM. ∗, p < 0.05; ∗∗, p < 0.01 vs. wild type; N = 8 mice per genotype (two-way ANOVA with Tukey's post-hoc test). (D) *Ex vivo* lipolysis in explants of inguinal (left) and epididymal (right) WAT from wild type and ALK7 mutant mice after HFD feeding induced by treatment with CL-316,243 (CL). Data are presented as mean ± SEM. ∗∗∗, p < 0.001; ∗∗∗∗, p < 0.0001; N = 5 mice per genotype (two-way ANOVA with Tukey's post-hoc test).

Fasting plasma glucose levels were comparable between the different mouse strains under both chow and HFD (Figure 3A,B). Alk7 KO showed elevated fasting insulin levels, particularly under HFD (Figure 3A,B), in agreement with previous observations [13]. Fasting insulin was also increased in I195T mice but was not different from wild type levels in I482V and N150H mice (Figure 3A,B). Glucose tolerance was within the normal range for all mutant mouse lines under normal chow diet (Figure 3C), but differences were found under HFD, with Alk7 KO mice showing signs of glucose intolerance compared to wild type (Figure 3D). Both Alk7 KO and I195T mice displayed reduced insulin sensitivity, particularly under HFD (Figure 3E,F), while I482V and N150H were not significantly different from wild type mice (Figure 3E,F). Reduced whole-body insulin sensitivity under HFD in Alk7-null mice has been attributed to fat accumulation in the liver [13]. In agreement with this I195T mice fed on HFD presented histological and biochemical features of liver steatosis (Figure 4A,B).

Whole-body lipolysis was assessed by determining glycerol plasma levels after injection of the specific Adrb3 agonist CL-316,243 (herein referred to as CL) in mice kept under normal diet or HFD. Alk7 KO, I195T and I482V mice showed enhanced whole-body lipolysis compared to wild type and N150H mice under HFD, with Alk7 KO displaying the highest level of the three (Figure 4C). Glycerol levels under normal diet kept under normal ranges for all lines of mice (Figure 4C). Adipose tissue lipolysis was measured *ex vivo* in explants from inguinal and epididymal WAT from HFD fed animals under both basal and CL-stimulated conditions. Alk7 KO mice displayed elevated levels of adipose tissue lipolysis in both fat depots under basal conditions (Figure 4D), while both Alk7 KO and I195T mice showed significantly elevated adipose tissue lipolysis upon CL stimulation (Figure 4D).

**Figure 5:**
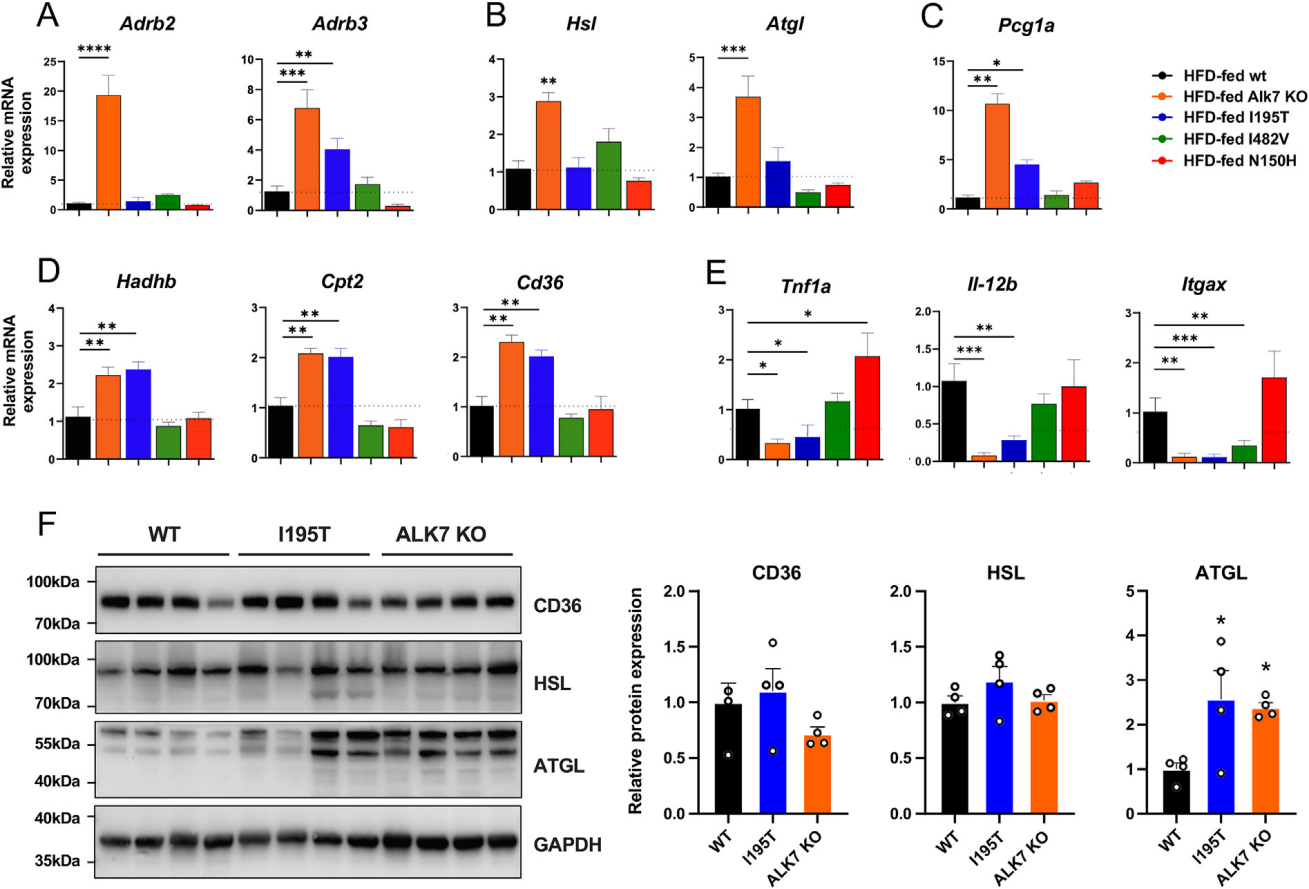
**Gene expression analysis in epididymal WAT of wild type and ALK7 mutant mice after high-fat diet feeding**. (A–E) mRNA levels assessed by quantitative RT-PCR for adrenergic receptors (A), lipolysis genes (B), mitochondria biogenesis master regulator *Pcg1a* (C), beta-oxidation genes (D), and inflammation genes (E). Data are presented as mean ± SEM of relative mRNA levels (normalized to wt levels). ∗, p < 0.05; ∗∗, p < 0.01; ∗∗∗, p < 0.001; ∗∗∗∗, p < 0.0001; N = 5 mice per genotype (one-way ANOVA with Tukey's post-hoc test). (F) Protein expression of CD36, HSL and ATGL in whole tissue extracts of epididymal WAT from wild type and ALK7 mutant mice after high-fat diet feeding analyzed by Western blotting. Data are presented as mean ± SEM relative to GAPDH levels (normalized to WT levels). ∗, p < 0.05; N = 4 mice per genotype (one-way ANOVA with Tukey's post-hoc test).

Expression of mRNAs encoding adrenergic receptors Adrb2 and Adrb3 was significantly increased in epididymal fat of Alk7 KO mice compared to wild type, and increased expression of *Adrb3* was also observed in epididymal adipose tissue from I195T mice (Figure 5A). Expression of lipolysis genes *Hsl* (encoding hormone sensitive lipase) and *Atgl* (adipose triglyceride lipase) was also increased in Alk7 KO but not in mice carrying *Acvr1c* variants (Figure 5B). Expression of mRNA encoding PCG-1α, a master regulator of mitochondrial biogenesis, as well as several genes involved in fatty acid beta-oxidation, including *Hadhb*, *Cpt2* and *Cd36*, were elevated in Alk7 KO and to a lower degree in I195T mice, but not in the other strains (Figure 5C,D). Finally, expression of several markers of adipose tissue inflammation, including *Tnf1α*, *Il-12b* and *Itgax* mRNAs, was decreased in Alk7 KO and I195T mice but not in N150H mice (Figure 5E). I482V mice showed reduced expression of *Itgax*, but other inflammation markers were within the normal range (Figure 5E). At the protein level, we confirmed the increased expression of ATGL in adipose tissue of Alk7 KO mice and also detected elevated levels in I195T mice (Figure 5F). Protein levels of HSL and CD36 were not statistically significant between these strains (Figure 5F). We note that HSL is principally regulated by phosphorylation and significant changes in basal protein levels are not expected.

Together these results confirm previous studies on Alk7 KO mice and indicate similar, though not identical, metabolic alterations in I195T mice, a milder effect of the I482V variant and no effect of the N150H variant which remained close to wild type in all measures investigated.

### 3.3 Mice heterozygous for the I195T variant display normal weight gain and fat accumulation under high-fat diet

**Figure 6:**
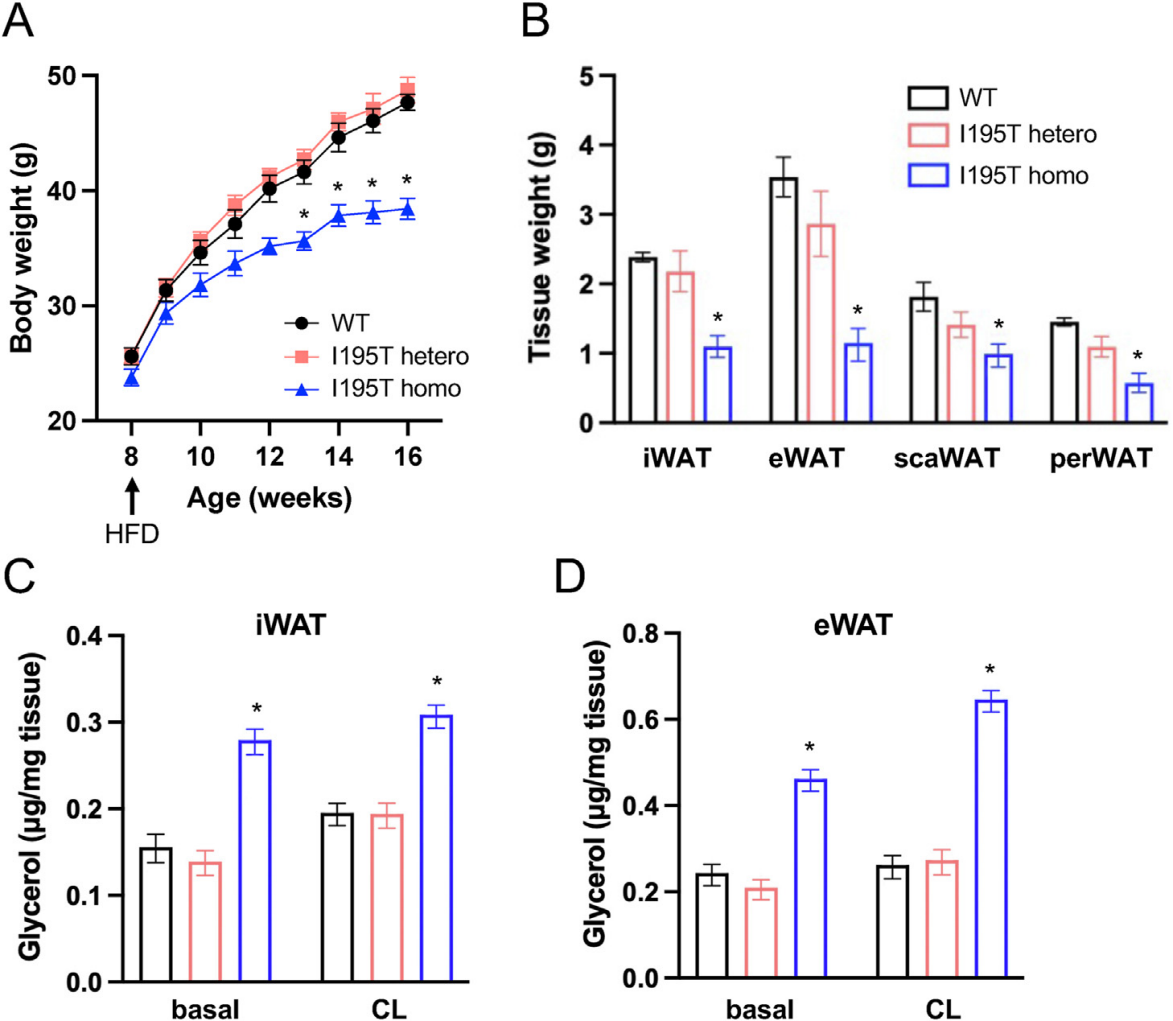
**Comparison of body weight, fat accumulation and lipolysis between homozygous and heterozygous I195T mice**. (A) Weight gain of wild type mice (WT), I195 heterozygous and homozygous mice fed on a normal chow diet (NCD) or high-fat diet (HFD) from 8 weeks of age (arrow). Data are presented as mean ± SEM. ∗, p < 0.05 vs. wt at 16 weeks (end of study); N = 10 mice per genotype (two-way ANOVA with Tukey's post-hoc test). (B) Wet-weight of inguinal (iWAT), epididymal (eWAT), scapular (scaWAT) and perirenal (perWAT) adipose tissue in wild type (WT), I195 heterozygous and homozygous mice after HFD feeding. Data are presented as mean ± SEM. ∗, p < 0.05. N = 5 mice per genotype (one-way ANOVA with Tukey's post-hoc test). (C, D) *Ex vivo* lipolysis in explants of inguinal (C) and epididymal (D) WAT from wild type (black), I195 heterozygous (red) and homozygous (blue) mice after HFD feeding induced by treatment with CL-316,243 (CL). Data are presented as mean ± SEM. ∗, p < 0.05; N = 5 mice per genotype (two-way ANOVA with Tukey's post-hoc test).

Koprulu et al. (2021) identified the I195T *ACVR1C* variant by testing the cumulative burden of rare gene variants affecting fat distribution in 184,246 individuals from the UK Biobank [8]. All individuals that carried the I195T variant were heterozygous for this allele. We therefore investigated whether this variant may affect fat accumulation when present in heterozygous form in mice. Wild type, I195T heterozygous and homozygous male mice were placed on HFD at 8 weeks of age for additional 8 weeks. Again, mice homozygous for I195T gained significantly less weight than wild type mice (Figure 6A). However, I195T heterozygous mice were indistinguishable from wild type (Figure 6A). Inguinal, epididymal, scapular and perirenal WAT depots were all significantly smaller in I195T homozygotes compared to wild type after 8 week HFD (Figure 6B), but these depots remained unchanged in I195T heterozygous mice (Figure 6B). Analysis of *ex vivo* lipolysis in explants from inguinal and epididymal WAT from HFD fed animals under both basal and CL-stimulated conditions revealed, as before, significantly increased lipolysis in WAT from I195T homozygous mice (Figure 6C,D). However, WAT *ex vivo* lipolysis was unchanged in heterozygous mice (Figure 6C,D). Together, these results indicate that in mice of C57BL/6J genetic background, the I195T *ACVR1C* variant is recessive and can only affect fat accumulation and diet-induced obesity when present in homozygous form.

### 3.4 All human *ACVR1C* missense variants impair ALK7 signaling to Smad2/3 proteins, although N150H only at low ligand concentrations

**Figure 7:**
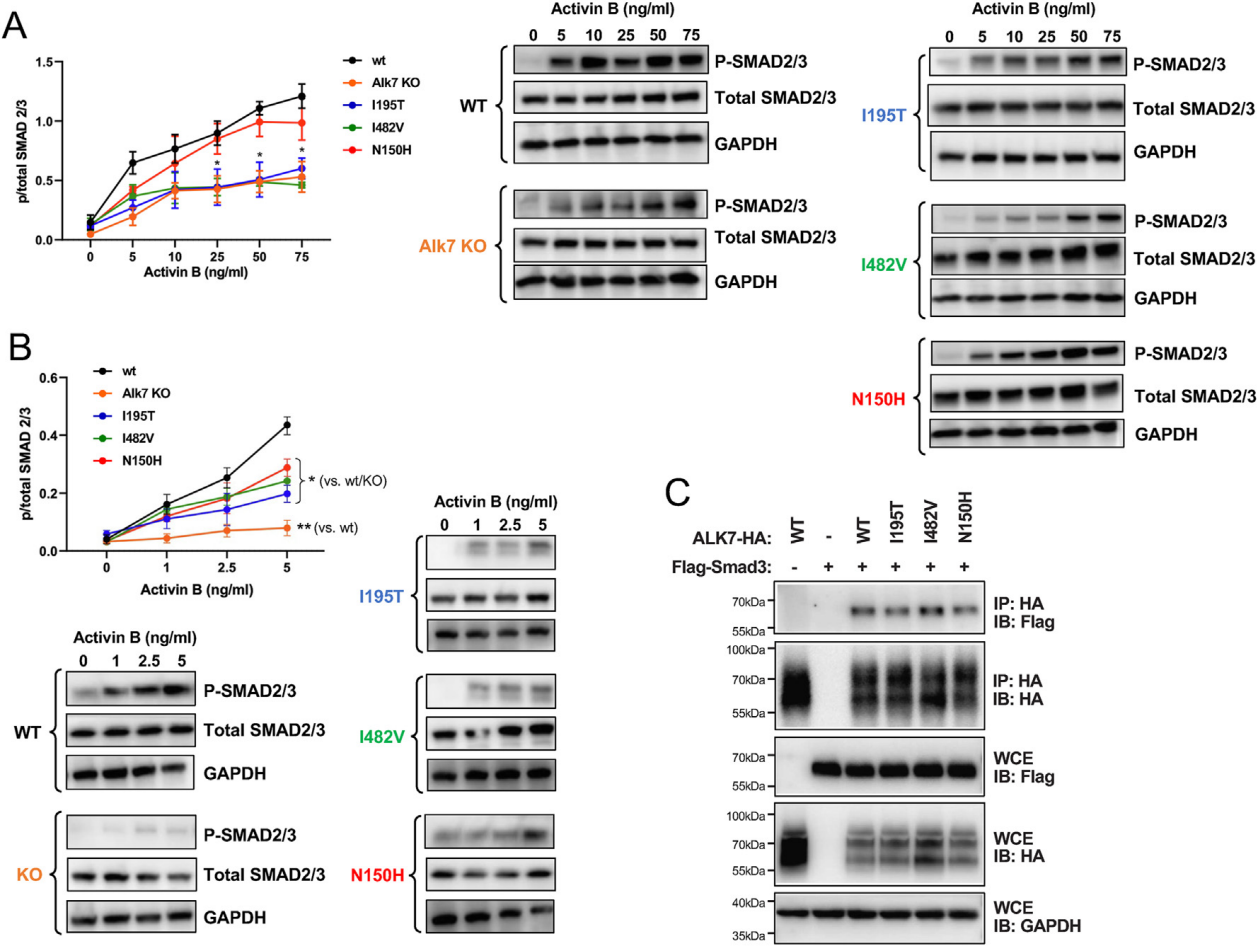
**ALK7 signaling to Smad2/3 proteins in cultured adipocytes from wild type and ALK7 mutant mice**. (A) Representative Western blots and quantification of Smad2/3 phosphorylation in cultured adipocytes from inguinal WAT of the indicated genotypes following treatment with activin B (0–75 ng/ml). The antibody used that does not distinguish between the two phospho-Smads. Total Smad2/3 and GAPDH (glyceraldehyde phosphate dehydrogenase) were probed as loading controls. Quantifications are presented as mean ± SEM of relative Smad2/3 phosphorylation (phospho/total). ∗, p < 0.05 vs. wt; N = 3 experiments each performed in triplicate (two-way ANOVA with Tukey's post-hoc test). (B) Idem (A) but for lower concentrations of activin B (0–5 ng/ml). ∗, p < 0.05 vs. wt and/KO; ∗∗, p < 0.01 vs. wt; N = 3 experiments each performed in triplicate (two-way ANOVA with Tukey's post-hoc test). (C) Co-immunoprecipitation of HA-tagged wild type (WT) and ALK7 variants with Flag-tagged Smad3 in transfected HEK293 cells. IP, immunoprecipitation; IB, immunoblotting, WCE, whole cell lysate.

To assess the signaling capabilities of the three missense variants in *Acvr1c*, we established cultures of stromal vascular fraction (SVF) from inguinal WAT and induced adipogenic differentiation of precursors into mature adipocytes following established protocols [5,12]. Adipocyte cultures were then stimulated with increasing doses of activin B and phosphorylation of Smad2/3, the main downstream mediators of ALK7 signaling, was investigated by Western blotting using an antibody that does not distinguish between the two phospho-Smads. As expected, adipocytes derived from Alk7 KO mice displayed significantly lower levels of Smad2/3 phosphorylation in response to activin B compared to wild type cultures (Figure 7A). The remaining Smad2/3 phosphorylation observed in these cells can be attributed to activation of ALK4 by activin B, which is also expressed in adipocyte cultures, although at lower levels than ALK7 [12]. Adipocytes derived from I195T and I482V mice were also largely impaired in their ability to respond to activin B (Figure 7A). In contrast, N150H adipocytes showed responses similar to wild type, particularly at concentrations above 10 ng/ml (Figure 7A). Analysis of Smad2/3 phosphorylation at lower activin B concentrations (below 5 ng/ml) revealed a range of graded responses that was in good agreement with the relative strength of the metabolic phenotypes induced by the different variants. At these low doses of ligand, Alk7 KO cells showed no significant response, followed by I195T, I482V and N150H (Figure 7B). Interestingly, the response of N150H cells was still significantly lower than wild type below 5 ng/ml (Figure 7B), indicating that this mutation does cause a functional impairment at the molecular level, although not sufficient to alter metabolic functions in knock-in mice. Inefficient Smad phosphorylation may arise from either reduced kinase activity or diminished substrate binding to the receptor kinase domain. To distinguish between these two possibilities, we conducted co-immunoprecipitation experiments in HEK293 cells expressing epitope tagged Smad3 and ALK7 constructs. Specific binding of Smad3 to ALK7 could be detected at comparable levels in all variants (Figure 7C), indicating that the mutations do not significantly affect the recruitment of substrate to the kinase domain and are therefore more likely to impact kinase activity.

**Figure 8:**
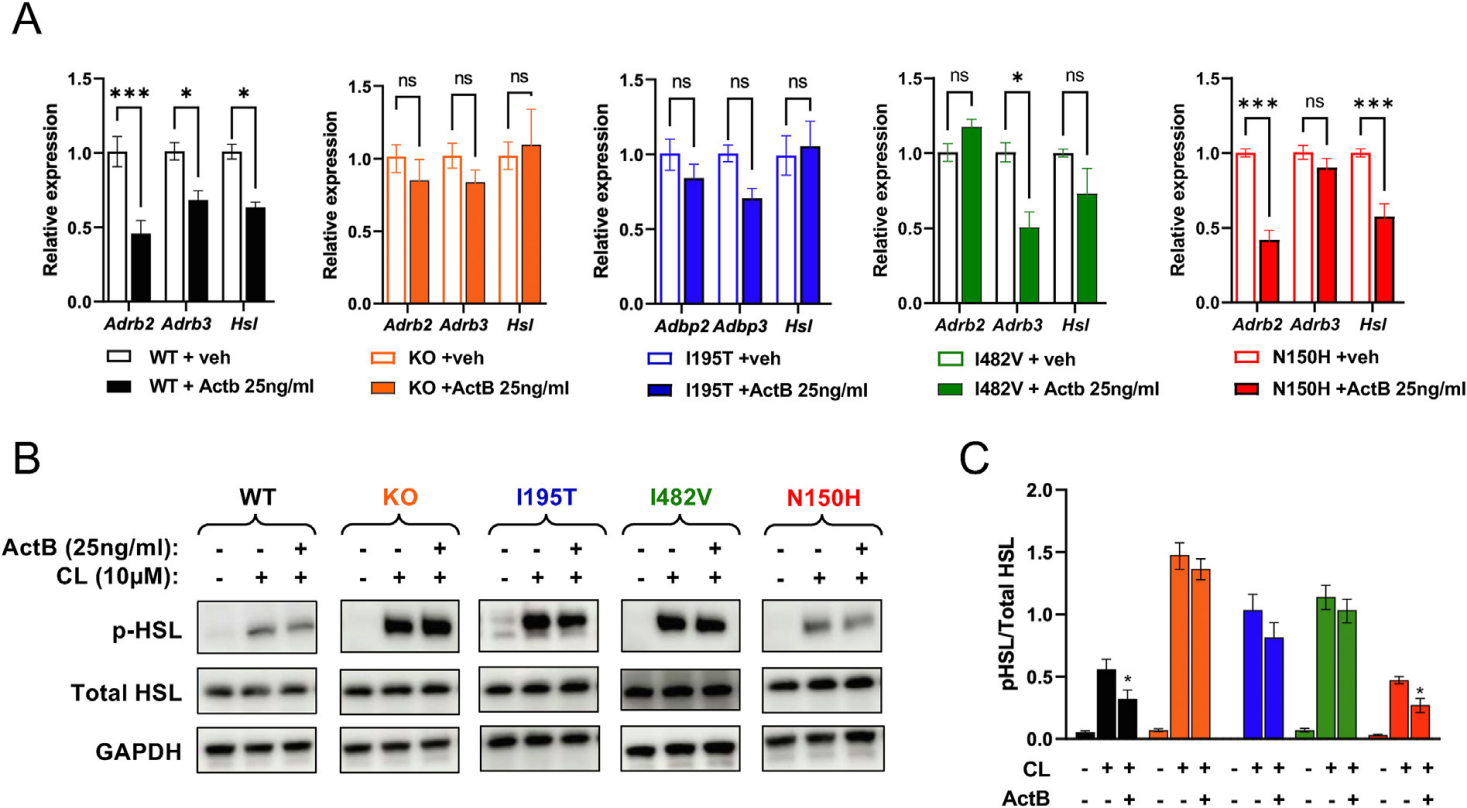
**ALK7 signaling to downstream target genes *Adrb2*, *Adrb3* and *Hsl* in cultured adipocytes from wild type and ALK7 mutant mice**. (A) Results of mRNA levels assessed by quantitative RT-PCR for Adrb2, Adrb3 and Hsl in cultured adipocytes from inguinal WAT of the indicated genotypes following treatment with 25 ng/ml activin B. Data are presented as mean ± SEM of relative mRNA levels (normalized to wt levels). ∗, p < 0.05; ∗∗∗, p < 0.001; N = 3 experiments each performed in triplicate (two-way ANOVA with Tukey's post-hoc test). (B) Representative Western blots of HSL (hormone-sensitive lipase) phosphorylation in cultured adipocytes from inguinal WAT of the indicated genotypes following treatment with 25 ng/ml activin B and/or 10 μM CL-316,243 (CL). Total HSL and GAPDH were probed as loading controls. (C) Quantification of phosphorylation-HSL levels in cultured adipocytes treated with activin B and/or CL as indicated. Color codes for genotypes are as in panel (A). Data are presented as mean ± SEM of relative HSL phosphorylation (phospho/total). ∗, p < 0.05 vs. wt; N = 3 experiments each performed in triplicate (two-way ANOVA with Tukey's post-hoc test).

Previous studies have shown that activin B can suppress adrenergic signaling in adipocytes through ALK7 [4]. In agreement with this, the levels of mRNAs encoding Adrb2, Adrb3 and Hormone sensitive lipase (HSL) were all reduced in response to 25 ng/ml activin B in cultures of wild type adipocytes (Figure 8A). Expression of *Adrb2* and *Adrb3* and *Hsl* mRNAs remained unchanged in adipocytes derived from Alk7 KO and I195T mice treated with activin B (Figure 8A). At this concentration, activin B was able to reduce expression of *Adrb3* mRNA in adipocytes derived from I482V mice, while the levels of Adrb2 and Hsl mRNAs were not significantly affected in this strain (Figure 8A). In N150H adipocytes, 25 ng/ml activin B reduced expression of *Adrb2* and *Hsl* mRNAs, while reduction of Adrb3 mRNA levels was not statistically significant (Figure 8A). Finally, activation of HSL, as assessed by phosphorylation of Ser^660^, in response to adrenergic stimulation with CL was examined in cultured adipocytes treated with 25 ng/ml activin B. Robust phosphorylation of HSL was observed in adipocytes after treatment with CL (Figure 8B,C). As previously reported [4], activin B could partially suppress CL-induced HSL phosphorylation in adipocytes expressing wild type ALK7 (Figure 8B,C), in line with its ability to suppress adrenergic signaling. Higher levels of HSL phosphorylation were induced by CL in adipocytes derived from Alk7 KO, I195T and I482V mice, which remained largely unaffected by activin B (Figure 8B,C). In contrast, N150H adipocytes behaved similarly to wild type and responded to activin B with reduced HSL Ser^660^ phosphorylation (Figure 8B,C).

Thus, all *Acvr1c* variants, including N150H, show some level of functional impairment at the molecular level, but only the two most severely affected, namely I195T and I482V, produce metabolic phenotypes consistent with ALK7 loss of function in knock-in mice.

## 4 Discussion

A number of different missense and non-coding variants have been identified in the human *ACVR1C* gene in several recent studies [[6], [7], [8]]. Of the missense variants, N150H, I195T and I482V have been consistently reported to correlate with reduced WHR/BMI, a measure of body fat distribution in humans. However, whether these variants affect the function of ALK7 in adipocytes and have a direct, causative effect on fat accumulation has been unknown. The goal of the present study was to address this by introducing the three variants in the mouse *Acvr1c* locus through homologous recombination and assess their impact on ALK7 signaling, adipose tissue function and diet-induced obesity. The results indicate that although all variants affect ALK7 signaling to varying degrees in mouse adipocytes, only I195T and I482V had effects on diet-induced obesity, fat accumulation and adipose tissue lipolysis that are consistent with a loss of ALK7 function. One limitation of the present study is the analysis of the mice at a fixed point in time. Additional information may be obtained by studying the role of the variants in the progression of metabolic disease over time.

We find that the degree to which the different variants affected these phenotypes correlates with their signaling strength. Although I195T was the variant that most closely resembled the null mutation, it still showed some significant differences, particularly in basal (unstimulated) lipolysis and the levels of expression of several genes important for adipose tissue function, such as *Adrb2*, *Hsl* and *Pcg1a*, which were all closer to wild type levels, suggesting that I195T is not a complete loss-of function mutation. Thus, ligand-stimulated Smad2/3 phosphorylation in adipocytes derived from I195T mice was lower, but still detectable, than in wild type adipocytes at low (<5 ng/ml) doses of ligand. In contrast, adipocytes from ALK7 knock-out mice were largely insensitive to low concentrations of activin B. In their previous study, Koprulu et al. compared the ability of wild type and I195T ALK7 variants to induce Smad phosphorylation and a luciferase reporter in transfected HEK cells [8]. While I95T was shown to be impaired when stimulated with GDF3, it was not when stimulated with activin B. We attribute this result to receptor overexpression and/or endogenous expression of other activin receptors in HEK cells, such as ALK4.

While N150H reduced ALK7 signaling only at low ligand concentrations, it had no apparent effects on metabolism, suggesting the existence of a threshold below which suboptimal signaling translates into altered adipose tissue function. The fact that some human carriers of this variant show WHR/BMI changes suggests a lower threshold in humans compared to mice. In this regard, one limitation of the present study is that *Acvr1c* variants were studied in mice of only one genetic background, namely C57Bl/6. This strain has been reported to carry a genetic predisposition to HFD-induced obesity, insulin resistance and nonalcoholic fatty liver disease [24,25]. It is possible that N150H may result in alterations in adipose tissue and fat accumulation in other mouse strains. Moreover, as the human is an outbred population, and not all individuals that carried *ACVR1C* variants showed changes in WHR/BMI, it may also be relevant to investigate the effects of these variants in outbred strains of mice. This could also explain why even the variant that showed the strongest effects, namely I195T, was largely inconsequential when present in heterozygous form in inbred C57Bl/6 mice, while all human carriers identified so far are heterozygous for this variant [8].

Unlike the human carriers of ACVR1C variants, both *Acvr1c* knock-out and I195T knock-in mice displayed insulin resistance and liver steatosis under a high fat diet (this study and refs. [10,13]). Development of insulin resistance in these mouse strains is likely due to high levels of circulating insulin levels and consistent with previously reported results [13]. It should be noted that, at the ages examined, the mutant mice did not show adverse metabolic phenotypes when kept on a normal diet; only when fed a high fat diet (60 % of calories from fat) did these mice develop insulin resistance and fatty liver. Although we do not have knowledge of the diets of the human carriers of ACVR1C variants, it is probably safe to speculate that they were not as high in fat content as the HFD used in our study. With regards to risks vis-a-vis benefits of ALK7 inhibition in therapeutic approaches to obesity, the fact that human carriers of ACVR1C variants were otherwise healthy is encouraging. Importantly, conditional ablation of ALK7 expression at adult stages in obese mice did not induce liver steatosis or insulin resistance [5], suggesting that the adverse metabolic phenotypes observed in constitutive *Acvr1c* mutants with impaired ALK7 signaling from birth may have a developmental component. A recent study using ALK7 blocking antibodies supports this notion [26].

Alignment of the protein sequences of mouse ALK4, ALK5 and ALK7 (Supplementary Fig. S3) reveals the degree of conservation of the amino acid residues altered by the three missense variants in *Acvr1c*. While Ile^195^ is conserved in both ALK4 and ALK5, Asn^150^ is present in ALK5 but replaced by Asp in ALK4, and Ile^482^ is replaced by Leu, a rather conservative change, in both ALK4 and ALK5 sequences. The crystal structure of the kinase domain of human ALK5 [27], whose sequence is over 87 % identical to that of ALK7, can be used as a guide to assess the possible structural or functional impact of the variants in the ALK7 kinase domain, for which a 3D structure is not yet available. ALK5 Ile^205^, homologous to ALK7 Ile^195^, is in close proximity to the binding site of FKBP12 at the top of the N-terminal lobe of the ALK5 kinase [27]. FKBP12 is a ubiquitous and multifunctional protein that has been shown to interact with the inactive state of the ALK5 kinase thereby inhibiting signaling in the absence of ligand [28,29]. Intriguingly, although all seven key residues in the ALK5 kinase that contact FKBP12 are conserved in ALK7 (Supplementary Fig. S3), the latter appears to interact only very weakly if at all with FKBP12 and the I195T variant has no effect on the interaction (Supplementary Fig. S4A). In the crystal structure of the ALK5 kinase, the Ile^205^ side chain is buried inside the core of the protein and in close proximity to other hydrophobic residues (Supplementary Fig. S4B), all of which are conserved in the ALK7 protein sequence (Supplementary Fig. S3). At this position, a hydrophilic side chain such as Thr would likely disrupt the hydrophobic core of this region and conceivably alter the conformation of the N-terminal lobe of the kinase rendering it less active. It may also affect the phosphorylation of residues targeted by the type II receptor in the glycine-serine rich (GS) domain, which is required for activation of the ALK7 kinase, or the Smad substrate binding sites in both the GS domain and L45 loop [30], all of which lie close to this area. With regards to Smad binding, however, our co-immunoprecipitation experiments between Smad3 and ALK7 suggests that Smad binding was not altered in the I195T mutant. The side chain of Leu^492^, the ALK5 kinase residue homologous of ALK7 Ile^482^, is also buried inside the structure and in close proximity to other hydrophobic residues, including Ile^303^, Phe^408^ and Leu^495^ (Supplementary Fig. S4C) which are also either conserved or replaced by residues of similar characteristics in the ALK7 kinase (Supplementary Fig. S3). Although also hydrophobic in character, Val is a much smaller residue than Ile and may thus affect the stability of the core of the C-terminal lobe of the kinase. In ALK5, Leu^492^ is at the bottom of the structure, further away from both the ATP and substrate binding sites, which is in line with the effects of this variant being much milder than those of I195T. Finally, Asn^160^, the ALK5 residue homologous of ALK7 Asn^150^, is in the juxtamembrane region of ALK5 and not present in the crystal structure of the kinase. It is thus more difficult to speculate about its possible function, also because most studies addressing the role of ALK5 juxtamembrane region have mainly focused on the GS domain. An earlier study investigated the function of 30 amino acids long stretch in the ALK5 juxtamembrane region between the transmembrane and GS domains which included Asn^160^ [31]. A deletion mutant of ALK5 lacking this region was still able to bind ligand in concert with the type II receptor TBR-II and transduce a signal leading to increased expression of extracellular matrix proteins plasminogen activator inhibitor-1 (PAI-1) and fibronectin, but was impaired in its ability to inhibit cell proliferation [31]. Interestingly, point mutations in either Ser^172^ or Thr^176^, both present in this region, were able to mimic the effects of the deletion, although it is unclear whether these sites are targets of phosphorylation. Thr^176^, but not Ser^172^, is conserved in ALK7 (i.e. Thr^166^) and located 16 residues downstream of Asn^150^. It is therefore conceivable that the N150H variant may alter the conformation of this region leading to a partial loss of ALK7 function affecting certain downstream pathways but not others. The very limited effects of this variant in mice are in agreement with it being significantly less critical for receptor activity.

In conclusion, our analysis of the physiological and biochemical effects of three missense variants in mouse *Acvr1c*, which in humans correlate with reduced fat deposition, revealed metabolic alterations of graded strength, including resistance to diet-induced obesity, that correlated with the extent to which each variant affected ALK7 signaling in adipocytes. These findings, together with recent studies using conditional mutant mice and ALK7 blocking antibodies [5,26] support the validity of ALK7 as a therapeutic target in human obesity and its metabolic consequences. A detailed understanding of the mechanisms by which each variant alters ALK7 function will require additional structural and biochemical studies; such knowledge may facilitate the design of pharmacological strategies that phenocopy their effects.

## Sources of funding

This work was supported by research grants to C.F.I. from 10.13039/501100007937Peking University, Chinese Institute for Brain Research, Beijing, 10.13039/501100002794Swedish Cancer Society (10.13039/501100002794Cancerfonden, contract nr. 222135Pj01H) and 10.13039/501100004359Swedish Research Council (Vetenskapsrådet, contract nr. 2020-01923_3); and a startup grant to M.X. from 10.13039/501100004359Swedish Research Council (Vetenskapsrådet, contract nr. 2021-01805).

## CRediT authorship contribution statement

**Pawanrat Tangseefa:** Investigation, Formal analysis. **Hong Jin:** Investigation. **Houyu Zhang:** Investigation. **Meng Xie:** Writing – review & editing. **Carlos F. Ibáñez:** Writing – review & editing, Writing – original draft, Supervision, Funding acquisition, Formal analysis, Conceptualization.

## Declaration of competing interest

The authors declare that they have no known competing financial interests or personal relationships that could have influenced the results reported in this paper.

## Data Availability

Data will be made available on request.
