# Supplementary material for: Human ACVR1C missense variants that correlate with altered body fat distribution produce metabolic alterations of graded severity in knock-in mutant mice
Source: Mol Metab. 2024 Feb 1;81:101890. doi: 10.1016/j.molmet.2024.101890 (PMC10863331; doi:10.1016/j.molmet.2024.101890)
Supplement: Multimedia component 1 [file mmc1.pdf]

Appendix to Tangseefa et al. “Human *ACVR1C* missense variants that correlate with altered body fat distribution produce metabolic alterations of graded severity in knock-in mutant mice”

Table of contents:

Animal Report I195T……………………………………..…………...page 2

Animal Report I482V…………………..…………………………...page 10

Animal Report N150H……………………………………………..….page 19

cyagen
We help you discover life

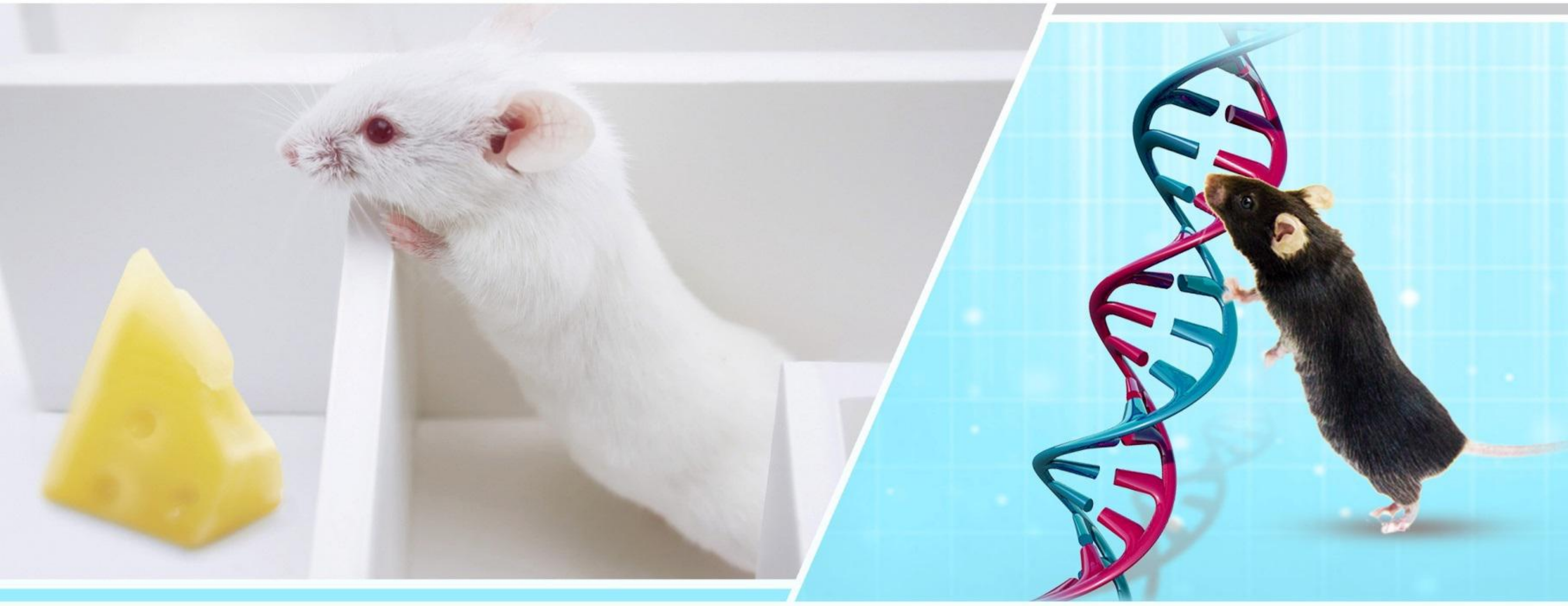

Animal Report

Quote: NTMCN-191031-CSC-02
Project: Mouse Acvr1c (p.I195T) Knockin

- Confidential -

1. Method

The gRNA to mouse Acvr1c gene, the donor oligo containing p.I195T (ATT to ACT) and p.R193= (AGG to CGT) mutations, and Cas9 were co-injected into fertilized mouse eggs to generate targeted knockin offspring. F0 founder animals were identified by PCR followed by sequence analysis, which were bred to wildtype mice to test germline transmission and F1 animal generation.

2. gRNA target sequence

gRNA (matching forward strand of gene): TGGTTCAAAGAACAATCGCAAGG

3. Diagram: Breeding Scheme

Parents

♂	**F0-9**	♀	**WT**

F1 mice

	**4 Males**		**1 Female**
D.O.B:	03-09-2020	D.O.B:	03-09-2020
Mouse ID:	4, 5, 6, 7	Mouse ID:	9

4. Genotyping Strategy

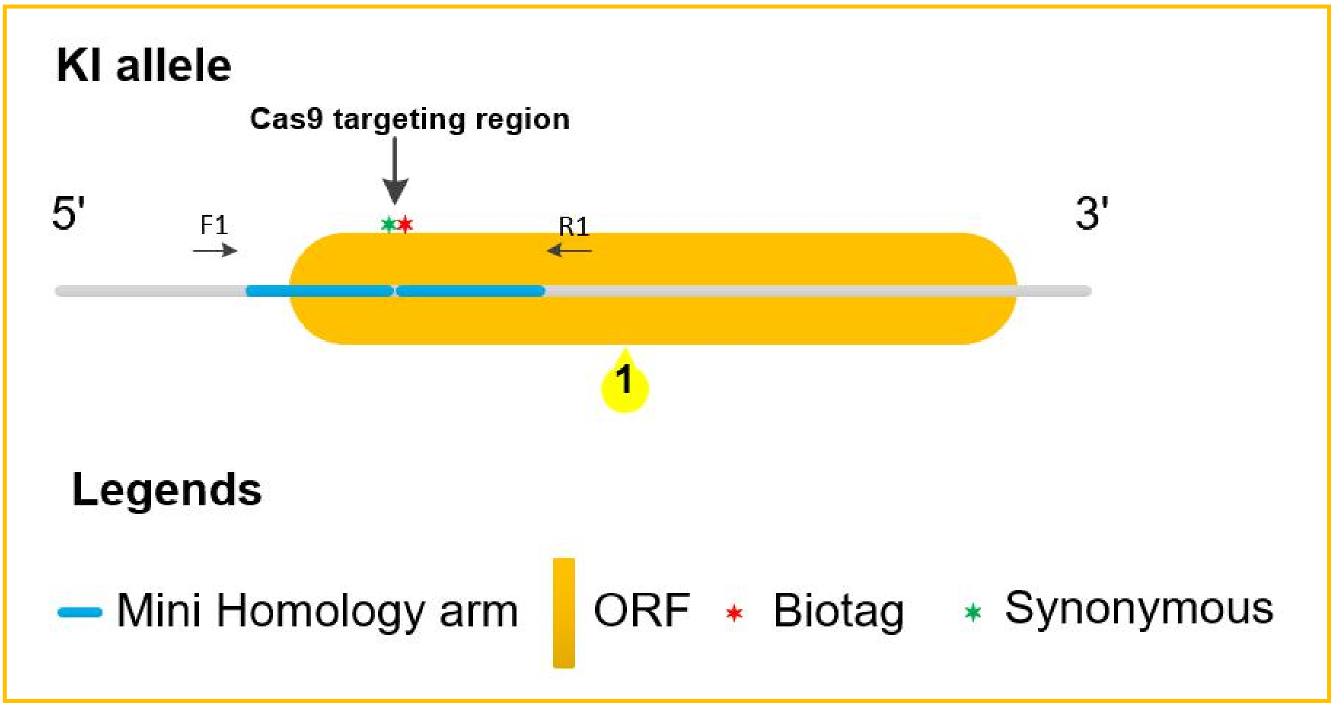

5. PCR Screening

PCR Primers (Annealing Temperature 60.0 ºC):

Forward primer (F1): 5’-AGTCAGACTGGCTTTGTGAGAAAAT-3’
Reverse primer (R1): 5’-AAGCCTGACCTTACCTTTGTTGT-3’

PCR Results:

F1 animals 4, 5, 6, 7 and 9 and other animals amplified by PCR, the PCR product will be used to sequencing confirmation.

Marker **Positive F1 from founder 9# (MT: 691 bp; WT: 691 bp)**

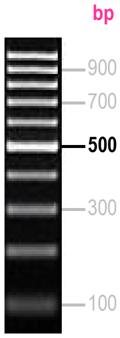

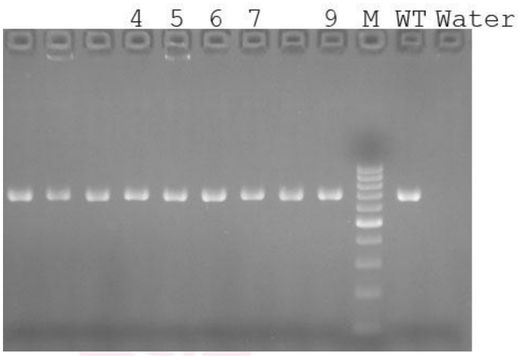

Note:

1) PCR was carried out in 50 µL volume for 33 cycles under standard conditions, with all two primers listed above added to each reaction.

2) Taq DNA polymerase used was LongAmp Taq DNA polymerase (NEB M0323V).

3) Two controls used in PCR genotyping are:

- Water control: No DNA template added.
- Wildtype control: 400 ng of mouse genomic DNA.

6. Sequencing Confirmation

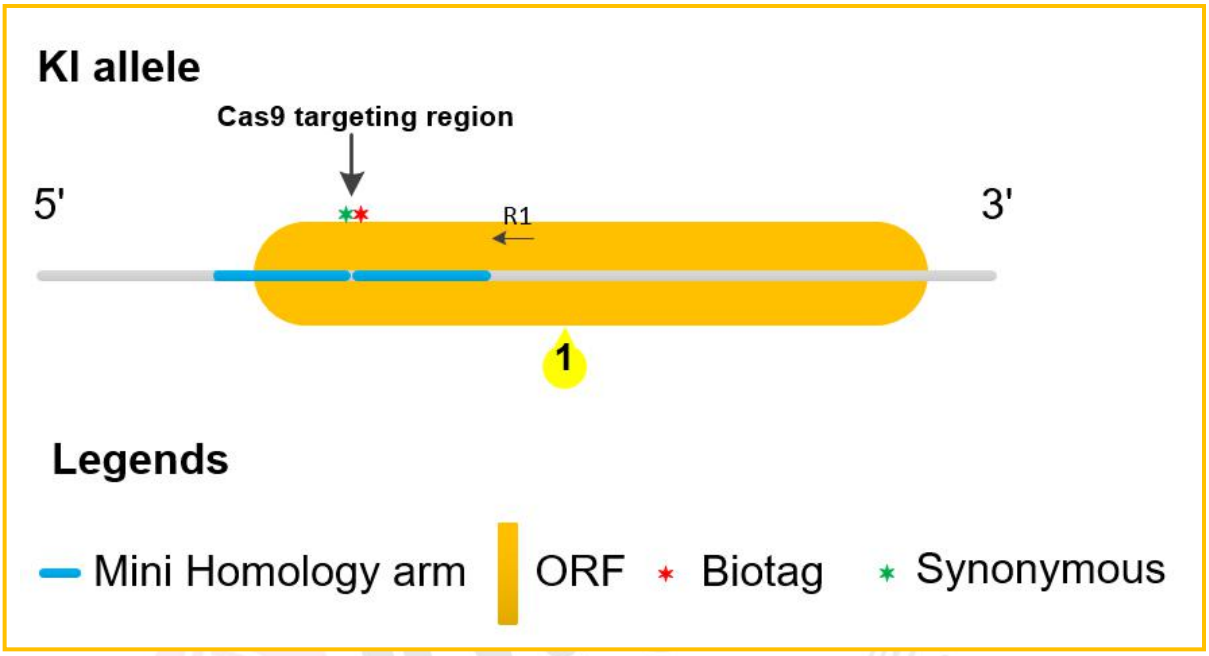

Sequencing Primer:

R1: 5’-AAGCCTGACCTTACCTTTGTTGT-3’

Sequencing Results:

F1 animals 4, 5, 6, 7 and 9 from founder 9# With p.I195T (ATT to ACT) mutation and silent mutation p.R193= (AGG to CGT)

Positive F1 from founder 9#

Mouse ID: 4

Wildtype：ACTAGGTCTGCCTCTCTTGGTTCAAAGAACAATCGCAAGGACAATTGTACTTCAAGAAATCGTAGGAAAAGGTCGGTTTG

Mutation：ACTAGGTCTGCCTCTCTTGGTTCAAAGAACAATCGCACGTACAACTGTACTTCAAGAAATCGTAGGAAAAGGTCGGTTTG

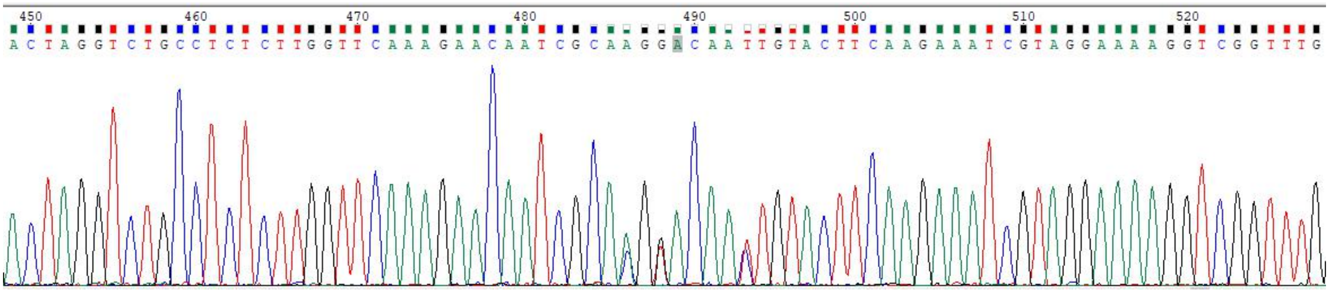

www.cyagen.com
Tel: 800-921-8930; Fax: 408-969-0336
2255 Martin Ave., Suite E. Santa Clara, CA 95050

7. Targeting Strategy

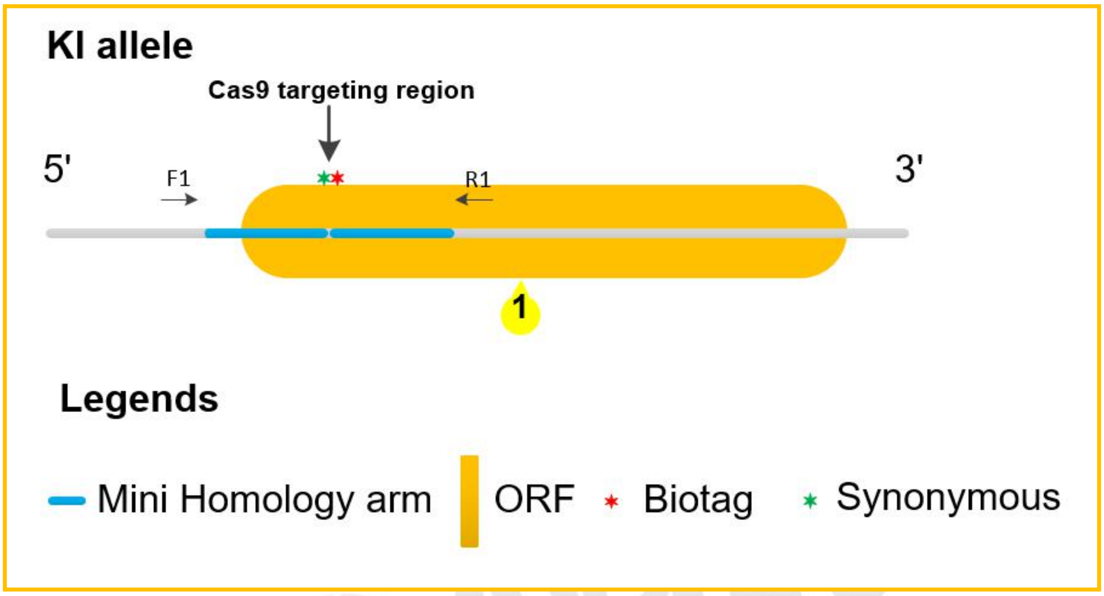

8. Cyagen Delivered

Heterozygous recombinant mice

9. Breeding and Genotyping strategy

Inter-cross heterozygous targeted mice to generate homozygous targeted mice

Primers:

F1: 5'-AGTCAGACTGGCTTTGTGAGAAAAT-3'
R1: 5'-AAGCCTGACCTTACCTTTGTTGT-3'
PCR product size 691 bp

The 691 bp PCR product will be used to sequencing confirmation.

Sequencing Primer:
F1: 5'-AGTCAGACTGGCTTTGTGAGAAAAT-3'
Sequencing Confirmation:
Homozygotes:
MT: ACTAGGTCTGCCTCTCTTGGTTCAAAGAACAATCGCACGTACAACTGTACTTCAAGAAATCGTAGGAAAAGGTCGGTTTG

Heterozygotes:
MT: ACTAGGTCTGCCTCTCTTGGTTCAAAGAACAATCGCACGTACAACTGTACTTCAAGAAATCGTAGGAAAAGGTCGGTTTG
WT: ACTAGGTCTGCCTCTCTTGGTTCAAAGAACAATCGCAAGGACAATTGTACTTCAAGAAATCGTAGGAAAAGGTCGGTTTG

Wildtype allele:
WT: ACTAGGTCTGCCTCTCTTGGTTCAAAGAACAATCGCAAGGACAATTGTACTTCAAGAAATCGTAGGAAAAGGTCGGTTTG

www.cyagen.com
Tel: 800-921-8930; Fax: 408-969-0336
2255 Martin Ave., Suite E. Santa Clara, CA 95050

10. PCR Conditions Attachment

10.1 DNA Extraction

➢ Method One:

We recommend that using TaKaRa MiniBEST Universal Genomic DNA Extraction kit (Ver.5.0_Code No. 9765) to gain high purity of genomic DNA.

a. Add 180 μL of Buffer GL, 20 μL of Proteinase K and 10 μL of RNase A per tail piece (2-5 mm) in a microcentrifuge tube. Be careful not to cut too much tail.

b. Incubate the tube at 56℃ overnight.

c. Spin in microcentrifuge at 12,000 rpm for 2 minutes to remove impurities.

d. Add 200 μL Buffer GB and 200 μL absolute ethyl alcohol with sufficient mixing.

e. Place the spin Column in a collection tube. Apply the sample to the spin and centrifuge at 12,000 rpm for 2 min. Discard flow-through.

f. Add 500 μL Buffer WA to the spin column and centrifuge at 12,000 rpm for 1 min. Discard flow-through.

g. Add 700 μL Buffer WB to the spin column and centrifuge at 12,000 rpm for 1 min. Discard flow-through. (Note: Make sure the Buffer WB has been premixed with 100% ethanol. When adding Buffer WB, add to the tube wall to wash off the residual salt.)

h. Repeat step g.

i. Place the spin Column in a collection tube and centrifuge at 12,000 rpm for 2 min.

j. Place the spin Column in a new 1.5ml tube. Add 50~200 μL sterilized water or elution buffer to the center of the column membrane and let the column stand 5min. (Note: Heating sterilized water or elution buffer up to 65℃ can increase the yield of elution.)

k. To elute DNA, centrifuge the column at 12,000 rpm for 2 min. To increase the yield of DNA, add the flow-through and/or 50~200 μL sterilized water or elution buffer to the center of the spin column membrane and let the column stand 5 min. Centrifuge at 12,000 rpm for 2 min.

l. Quantify to genomic DNA. Eluted genomic DNA can be quantified by electrophoresis or electrophoresis.

➢ Method Two:

A low-cost and sample method to gain rough genomic DNA.

a. Add 100 μL of tail digestion buffer per tail piece (2-5 mm) in a microcentrifuge tube. Be careful not to cut too much tail.

b. Incubate the tube at 56 ℃ overnight.

c. Incubate the tube at 98 ℃ for 13 minutes to denature the Proteinase K.

d. Spin in microcentrifuge at top speed for 15 minutes. Use an aliquot of supernatant straight from the tube (2 μL in a 50 μL reaction) for PCR.

Final concentration of tail digestion buffer:

➢ 50 mM KCl

➢ 10 mM Tris-HCl (pH 9.0)

- 0.1 % Triton X-100
- 0.4 mg/mL Proteinase K

10.2 PCR Mixture:

Component	x1	
Mouse tail genomic DNA	2	μl
Forward primer (10 μM)	2	μl
Reverse primer (10 μM)	2	μl
dNTPs (2.5 mM)	6	μl
5X LongAmp Taq Reaction	10	μl
LongAmp Taq DNA Polymerase	2	μl
ddH_2O	26	μl
Total	50	μl

10.3 Cycling Condition:

Step	Temp.	Time	Cycles
Initial denaturation	94 °C	3 min	
Denaturation	94 °C	30 s	33 x
Annealing	60 °C	30 s	
Extension	65 °C	50 s/kb	
Additional extension	65 °C	10 min	

Contact Us

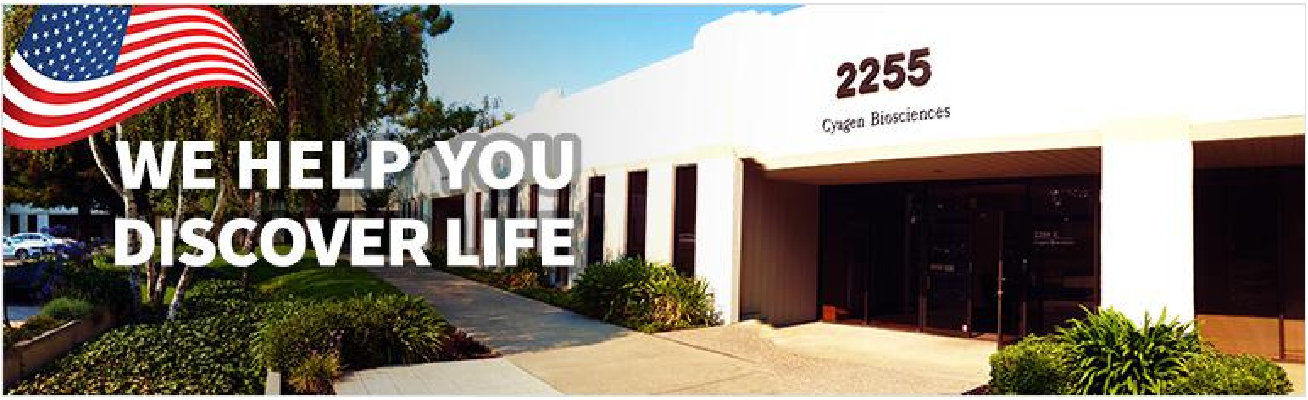

Thank you for choosing Cyagen US Inc as your research support. We sincerely hope that the mouse line generated will fulfill your expectation and will enable you to anticipate the next objective.

We also invite you to learn more about the positive impact that helping you discover life by visiting our website: www.cyagen.com.

- **North America and Europe (Headquarters)**

Cyagen US Inc.
2255 MARTIN AVE STE E
SANTA CLARA, CA 95050 – 2709, US.
Tel: US: 800-921-8930 (8-6pm PST)
+1 408-969-0306 (Int'l)
Europe: 800-793-45
+32-28085797 (Int'l)
Fax: 408-969-0336
Email: service@cyagen.com

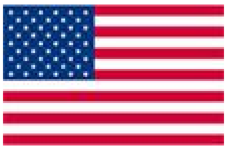

- **Asia**

Cyagen Biosciences (Guangzhou) Inc.
Building D, 3rd Floor, 3 Juquan Road, Science City
Guangzhou, 510663, China
Tel: 400-680-8038 or 86 20-28069059
Fax: +86-20-32290580
Email: info@cyagen.com

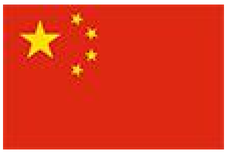

Cyagen Biosciences (Japan) Inc.
Room 4B, 1-20-10, Sugamo
Toshima ku, Tokyo 170-0002, Japan
Tel: 03-6304-1096
Fax: 03-6304-1098
Email: service@cyagen.jp

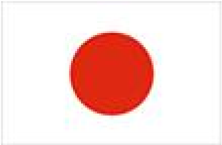

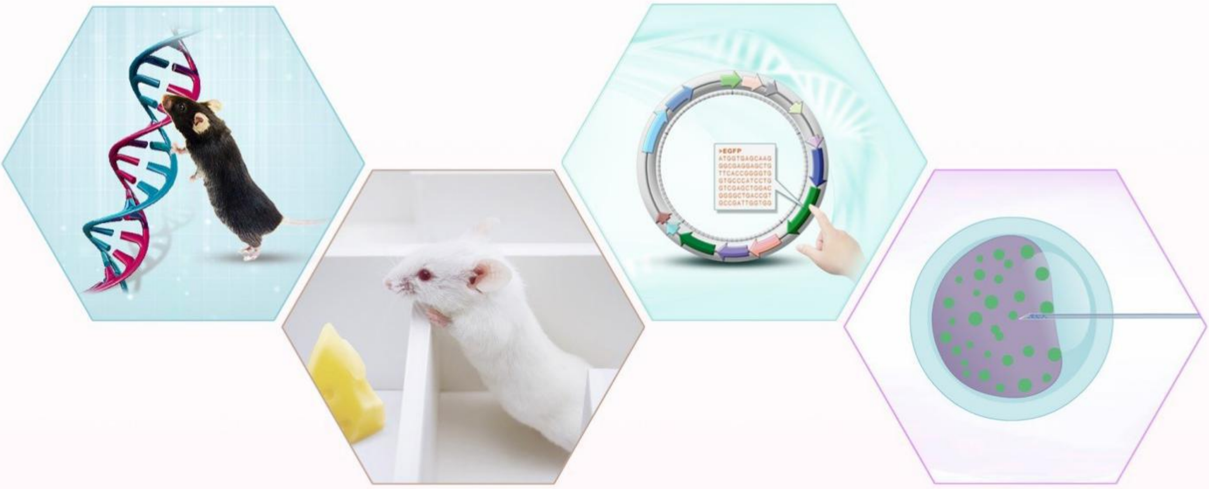

Animal Report

Quote: TKM-190328-CSC-02
Project: Mouse Acvr1c (I482V) Point Mutation

-Confidential-

1. Animal Generation

Targeted ES cell clone 8A9 was injected into C57BL/6 albino embryos, which were then re-implanted into CD-1 pseudo-pregnant females. Founder animals were identified by their coat color, their germline transmission was confirmed by breeding with C57BL/6 females and subsequent genotyping of the offspring. Two male and four female heterozygous targeted mice were generated from clone 8A9 as final deliverables for this project.

8A9 ESC, F1 mice

**2 Males ♂**		**4 Females ♀**	
D.O.B	12-10-2019	D.O.B	12-10-2019
Mouse ID	5	Mouse ID	7
D.O.B	12-12-2019	D.O.B	12-12-2019
Mouse ID	11	Mouse ID	13, 14, 15

1.1. Genotyping Strategy

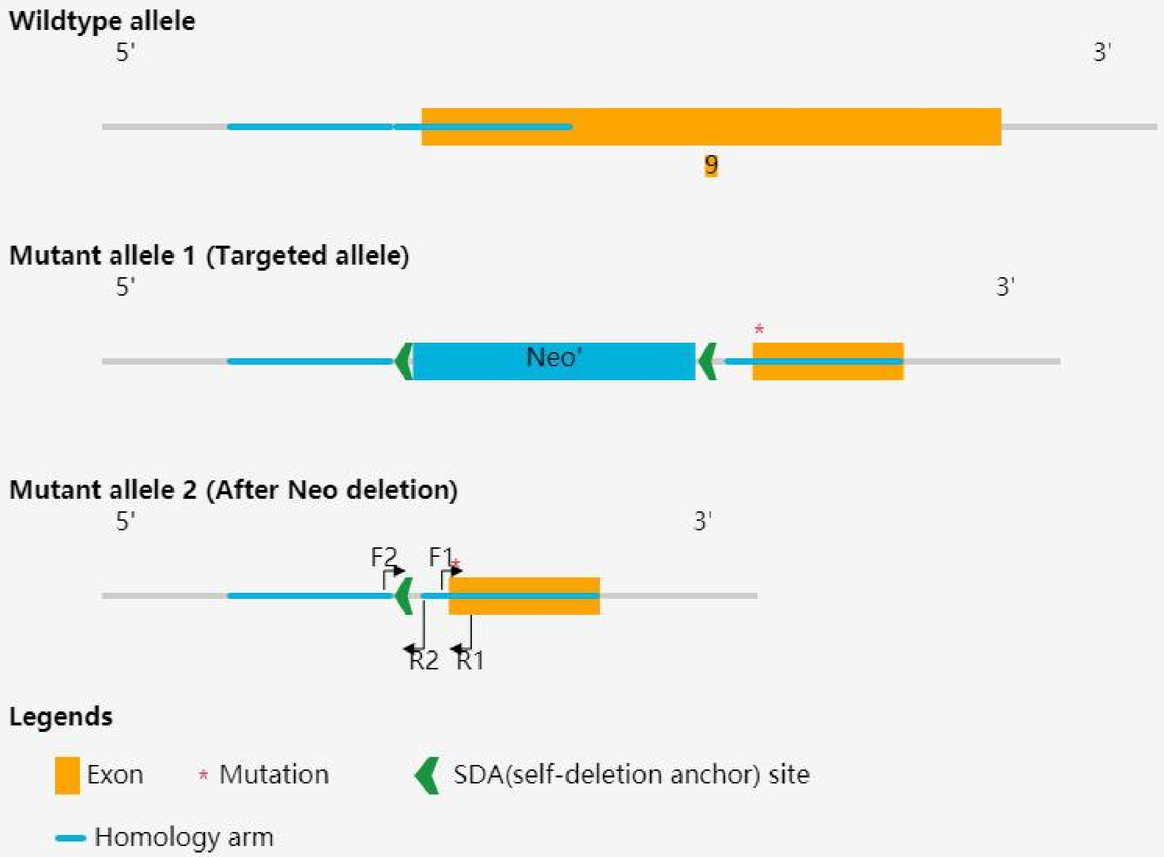

1.2. Cre-dele PCR

Primers for Cre-dele PCR:
F2: 5'-TATTTACCCTCAGACCATCGTTCG-3'
R2: 5'-TAAAAGGAGTCATGAGCTGTGGAA-3'

Expected PCR Product:
Wildtype: 165 bp
Targeted: 309 bp

Reaction Mix:

Component	x1
Mouse genomic DNA	1.5 µl
Forward primer (10 µM)	1.0 µl
Reverse primer (10 µM)	1.0 µl
Premix Taq Polymerase	12.5 µl
ddH_2O	9.0 µl
Total	25.0 µl

Cycling Condition:

Step	Temp.	Time	Cycles
Initial denaturation	94 °C	3 min	
Denaturation	94 °C	30 s	
Annealing	62 °C	35 s	33 x
Extension	72 °C	35 s	
Additional extension	72 °C	5 min	

Result

Six pups (5#, 7#, 11#, 13#, 14# and 15#) from clone 8A9 were identified positive by PCR screening for Cre-dele.

Cre-dele PCR, clone: 8A9 (WT: 165 bp; MT: 309 bp)

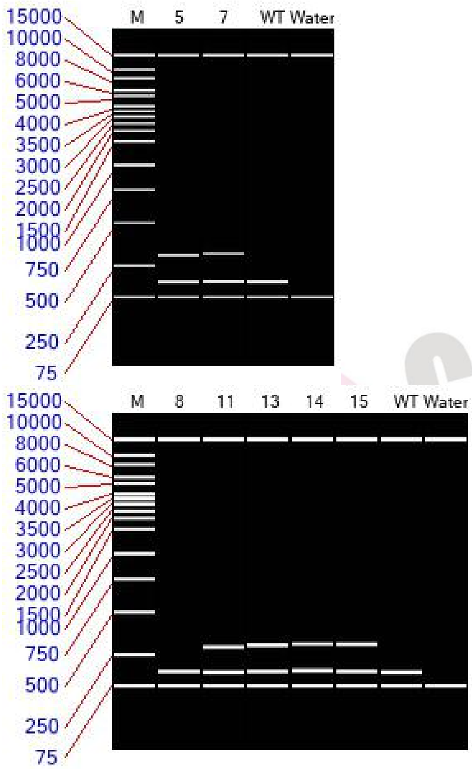

www.cyagen.com
Tel: 800-921-8930; Fax: 408-969-0336
2255 Martin Ave., Suite E. Santa Clara, CA 95050

1.3. The Sequencing Results

Primers for sequencing PCR:
F1: 5'-AGTTTCCTCGATCATCCCCTGAT-3'
R1: 5'-TAGCGTTATCTTTACATGCTGCCT-3'

Expected PCR Product:
Wildtype: 384 bp
Product Size: 384 bp

Primers for Sequencing:
R1: 5'-TAGCGTTATCTTTACATGCTGCCT-3'

Acvr1c_8A9_5#(R1)

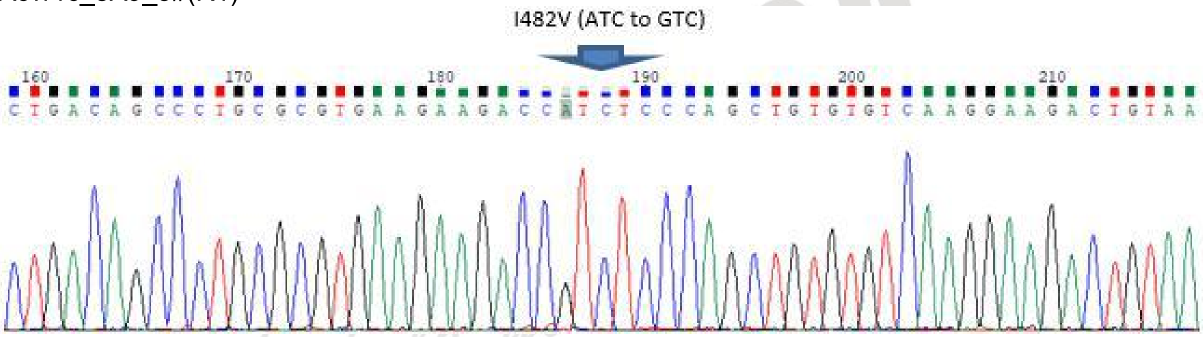

1.4. PCR Result:

Six pups (5#, 7#, 11#, 13#, 14# and 15#) from clone 8A9 were identified positive by PCR screening for Cre-dele, the positive pups were recomfirmed by PCR screening for Cre-dele.

1.5. Suggested Breeding and Genotyping Assay for Mice Generation

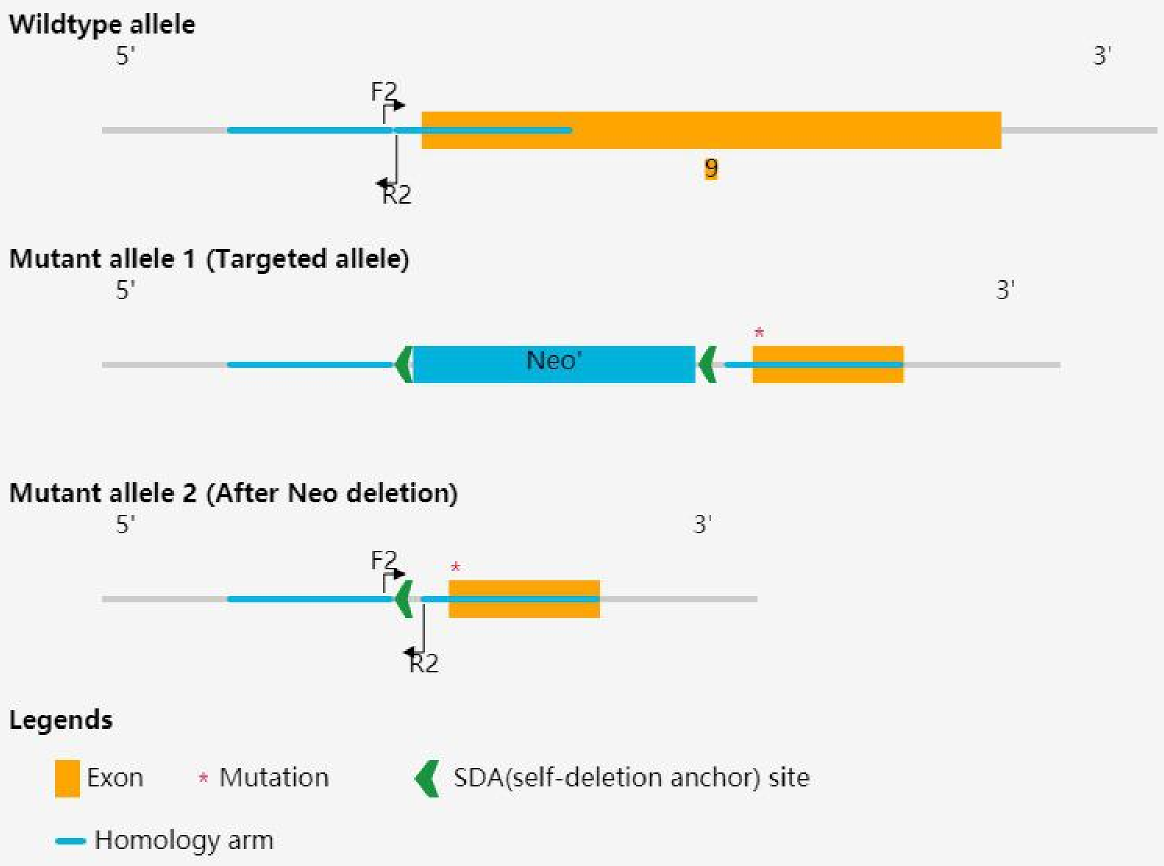

Step 1: Inter-cross heterozygous targeted mice to generate homozygous targeted mice

Primers for targeted allele:
F2: 5'-TATTTACCCTCAGACCATCGTTCG-3'
R2: 5'-TAAAAGGAGTCATGAGCTGTGGAA-3'

Wildtype: 165 bp
Homozygotes: 309 bp
Heterozygotes: 309 bp/165 bp

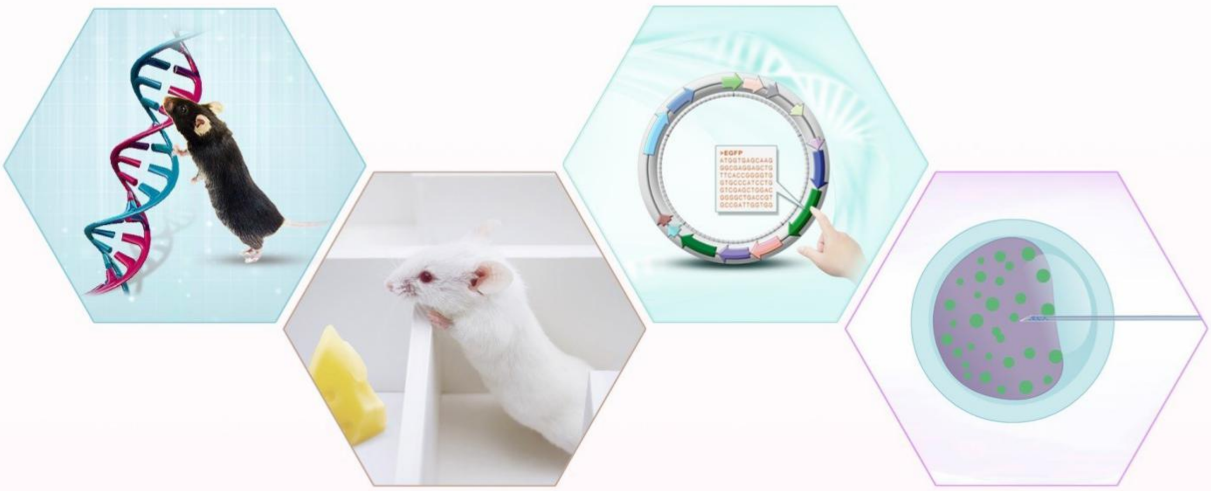

Animal Report

Quote: TKM-190328-CSC-01
Project: Mouse Acvr1c (N150H) Point Mutation

-Confidential-

1. Animal Generation

Targeted ES cell clone 1E7 was injected into C57BL/6 albino embryos, which were then re-implanted into CD-1 pseudo-pregnant females. Founder animals were identified by their coat color, their germline transmission was confirmed by breeding with C57BL/6 females and subsequent genotyping of the offspring. Two male and four female heterozygous targeted mice were generated from clone 1E7 as final deliverables for this project.

1E7 ESC, F1 mice

**2 Males ♂**		**4 Females ♀**	
D.O.B	10-11-2019	D.O.B	10-11-2019
Mouse ID	18, 21	Mouse ID	22, 23, 24, 25

cyagen
We help you discover life

1.1. Genotyping Strategy

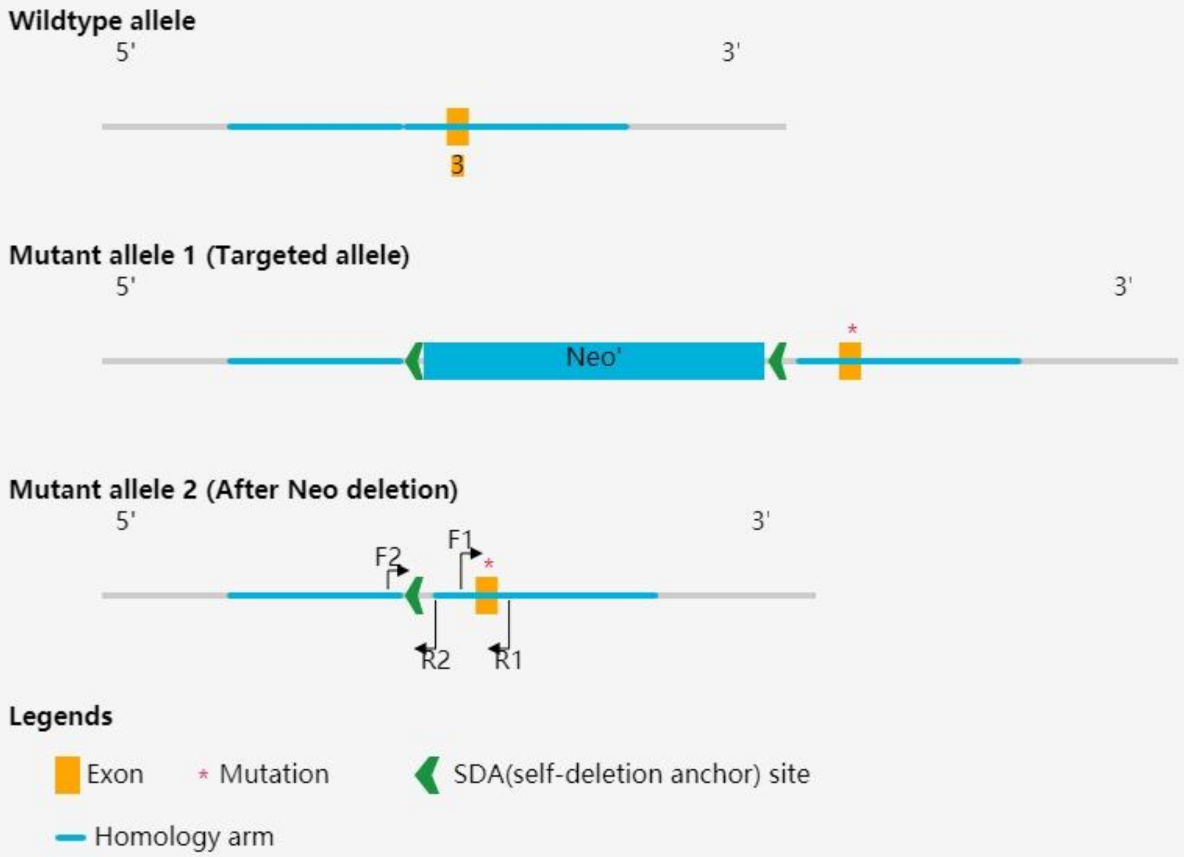

www.cyagen.com
Tel: 800-921-8930; Fax: 408-969-0336
2255 Martin Ave., Suite E. Santa Clara, CA 95050

1.2. Cre-dele PCR

Primers for Cre-dele PCR:
F2: 5'-GGTTTTCTCTAAGGACGAGACTGT-3'
R2: 5'-AATTCATTGCCAAAGGCCACAA-3'

Expected PCR Product:
Wildtype: 203 bp
Targeted: 347 bp

Reaction Mix:

Component	x1
Mouse genomic DNA	1.5 µl
Forward primer (10 µM)	1.0 µl
Reverse primer (10 µM)	1.0 µl
Premix Taq Polymerase	12.5 µl
ddH_2O	9.0 µl
Total	25.0 µl

Cycling Condition:

Step	Temp.	Time	Cycles
Initial denaturation	94 °C	3 min	
Denaturation	94 °C	30 s	
Annealing	62 °C	35 s	33 x
Extension	72 °C	35 s	
Additional extension	72 °C	5 min	

cyagen We help you discover life

Result

Six pups (18#, 21#, 22#, 23#, 24# and 25#) from clone 1E7 were identified positive by PCR screening for Cre-dele.

Cre-dele PCR, clone: 1E7 (WT: 203 bp; MT: 347 bp)

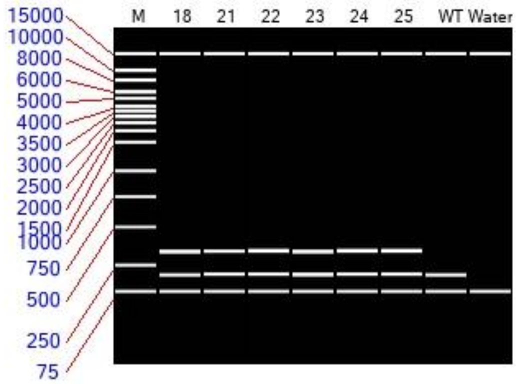

1.3. PCR Result:

Six pups (18#, 21#, 22#, 23#, 24# and 25#) from clone 1E7 were identified positive by PCR screening for Cre-dele, the positive pups were recomfirmed by PCR screening for Cre-dele.

1.4. Suggested Breeding and Genotyping Assay for Tissue-specific Knockout Mice Generation

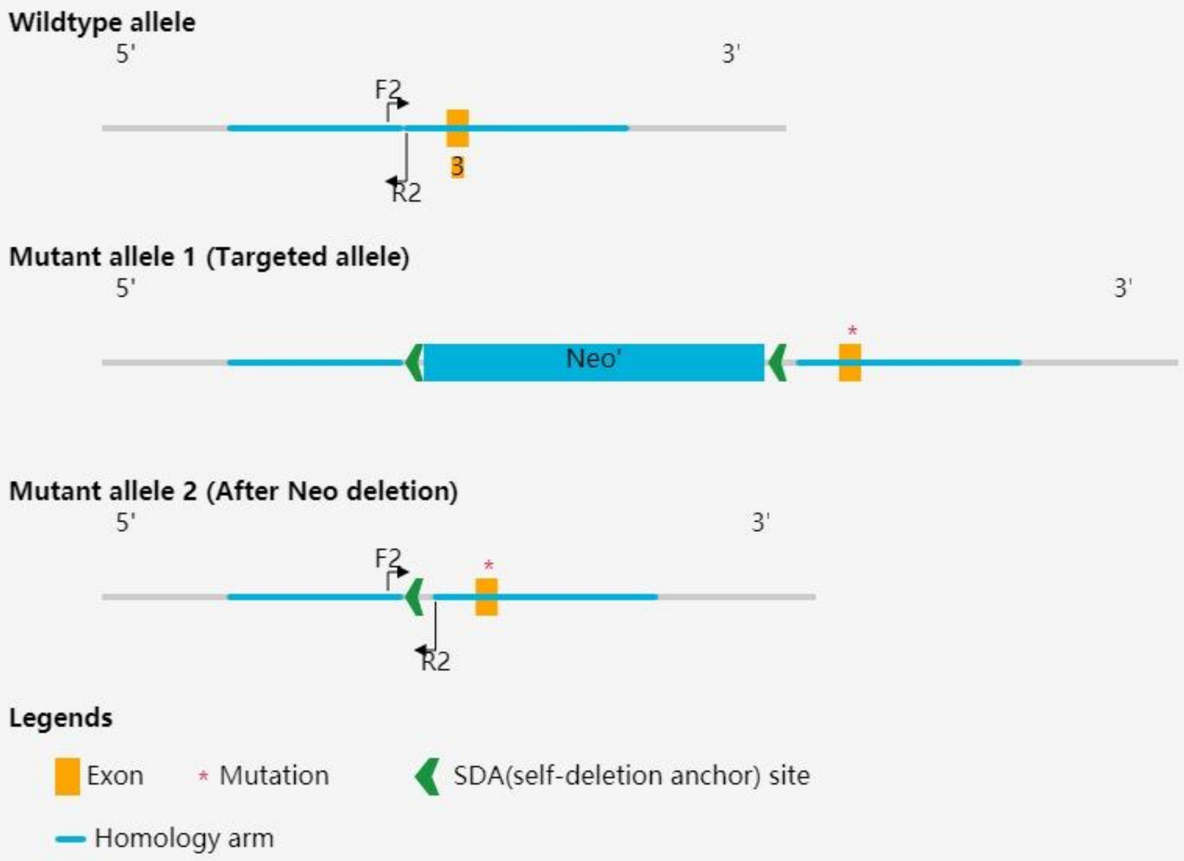

Step 1: Inter-cross heterozygous targeted mice to generate homozygous targeted mice

Primers for targeted allele:
F2: 5'-GGTTTTCTCTAAGGACGAGACTGT-3'
R2: 5'-AATTCATTGCCAAAGGCCACAA-3'

Wildtype: 203 bp
Homozygotes: 347 bp
Heterozygotes: 347 bp/203 bp
