# Supplementary material for: Human ACVR1C missense variants that correlate with altered body fat distribution produce metabolic alterations of graded severity in knock-in mutant mice
Source: Mol Metab. 2024 Feb 1;81:101890. doi: 10.1016/j.molmet.2024.101890 (PMC10863331; doi:10.1016/j.molmet.2024.101890)
Supplement: Multimedia component 2 [file mmc2.pdf]

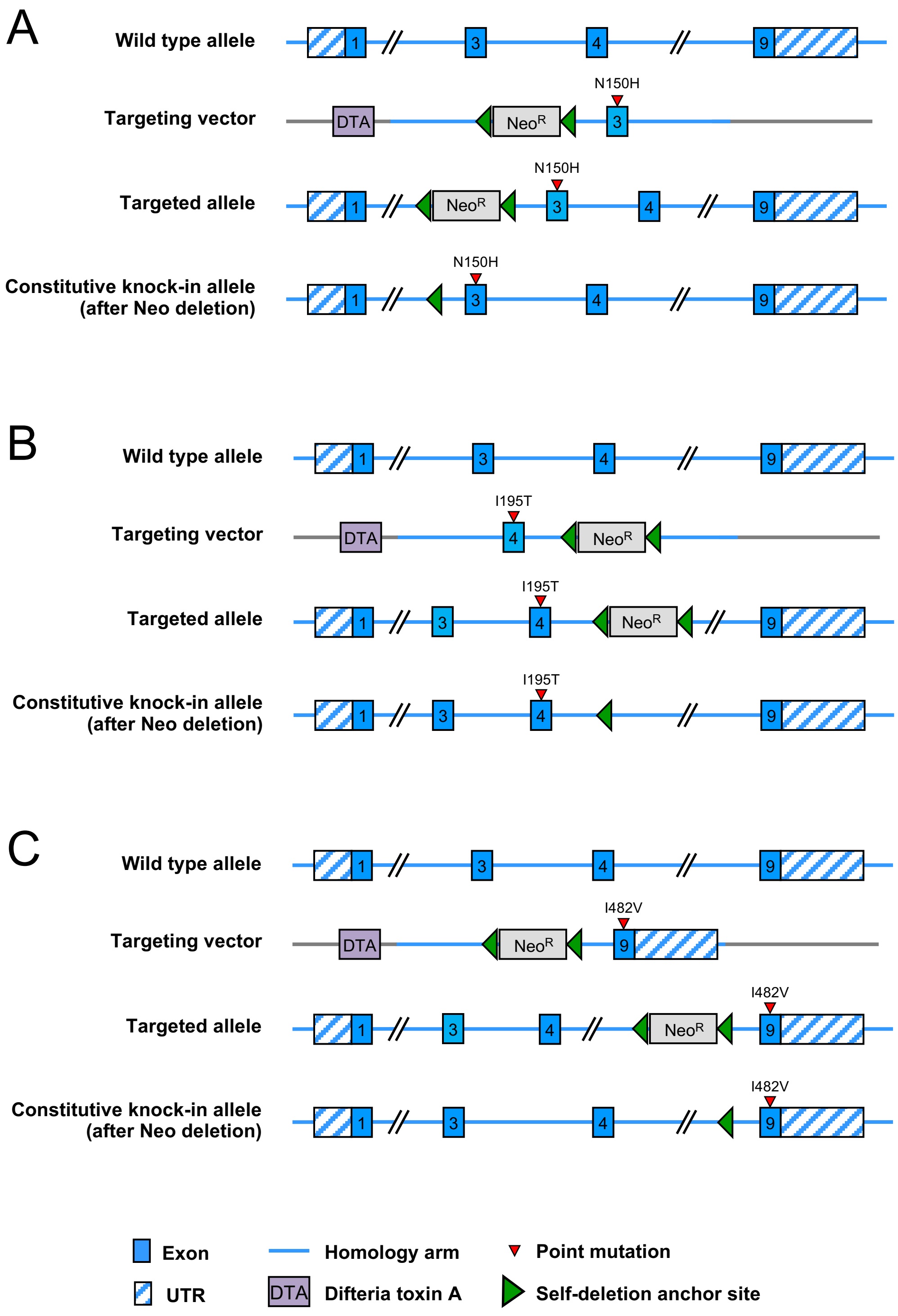

Figure S1. Generation of knock-in alleles in the mouse *Acvr1c* locus
(A) N150H was introduced in exon 3.
(B) I195T was introduced in exon 4.
(C) I482V was introduced in exon 9.
