# Supplementary material for: Human ACVR1C missense variants that correlate with altered body fat distribution produce metabolic alterations of graded severity in knock-in mutant mice
Source: Mol Metab. 2024 Feb 1;81:101890. doi: 10.1016/j.molmet.2024.101890 (PMC10863331; doi:10.1016/j.molmet.2024.101890)
Supplement: Multimedia component 3 [file mmc3.pdf]

```
mouse_alk7      MTPARGSALSLALLLVALAADLAAGLKCVCLLCDSSNFTCQTEGACWASVMLTNGKEQVI
rat_alk7        MTPASRSALSLALLLVALASDLAAGLKCVCLLCDSSNFTCQTEGACWASVMLTNGKEQVI
human_alk7      MTRALCSALRQALLLLAAAAELSPGLKCVCLLCDSSNFTCQTEGACWASVMLTNGKEQVI
                ** *  ***  ****:* *::*:.************************************

mouse_alk7      KSCVSLPELNAQVFCHSSNNVTKTECCFTDFCNNITLHLPTASPNAPRLGPTELTVVITV
rat_alk7        KSCVSLPELNAQVFCHSSNNVTKTECCFTDFCNNITLHLPTASPDAPRLGPTELTVVITV
human_alk7      KSCVSLPELNAQVFCHSSNNVTKTECCFTDFCNNITLHLPTASPNAPKLGPMELAIIITV
                ********************************************:**:*** **:::***

mouse_alk7      PVCLLSIAAMLTIWACQDRQCTYRKTKRHNVEEALAEYSLVNAGKTLKDLIYDATASGSG
rat_alk7        PVCLLSIAAMLTIWACQDRQCTYRKTKRHNVEEPLAEYSLVNAGKTLKDLIYDATASGSG
human_alk7      PVCLLSIAAMLTVWACQGRQCSYRKKKRPNVEEPLSECNLVNAGKTLKDLIYDVTASGSG
                ************:****.***:***.** ****.*:* .**************.******

mouse_alk7      SGLPLLVQRTIARTIVLQEIVGKGRFGEVWHGRWCGEDVAVKIFSSRDERSWFREAEIYQ
rat_alk7        SGLPLLVQRTIARTIVLQEIVGKGRFGEVWHGRWCGEDVAVKIFSSRDERSWFREAEIYQ
human_alk7      SGLPLLVQRTIARTIVLQEIVGKGRFGEVWHGRWCGEDVAVKIFSSRDERYWFREAEIYQ
                ************************************************** *********

mouse_alk7      TVMLRHENILGFIAADNKDNGTWTQLWLVSEYHEQGSLYDYLNRNIVTVAGMVKLALSIA
rat_alk7        TVMLRHENILGFIAADNKDNGTWTQLWLVSEYHEQGSLYDYLNRNIVTVAGMVKLALSIA
human_alk7      TVMLRHENILGFIAADNKDNGTWTQLWLVSEYHEQGSLYDYLNRNIVTMAGMIKLALSIA
                ************************************************:***:*******

mouse_alk7      SGLAHLHMEIVGTQGKPAIAHRDIKSKNILVKKCDTCAIADLGLAVKHDSIMNTIDIPQN
rat_alk7        SGLAHLHMEIVGTQGKPAIAHRDIKSKNILVKKCDTCAIADLGLAVKHDSIMNTIDIPQN
human_alk7      SGLAHLHMEIVGTQGKPAIAHRDIKSKNILVKKCETCAIADLGLAVKHDSILNTIDIPQN
                **********************************:****************:********

mouse_alk7      PKVGTKRYMAPEMLDDTMNLSIFESFKRADIYSVGLVYWEIARRCSVGGVVEEYQLPYYD
rat_alk7        PKVGTKRYMAPEMLDDTMNVNIFESFKRADIYSVGLVYWEIARRCSVGGLVEEYQLPYYD
human_alk7      PKVGTKRYMAPEMLDDTMNVNIFESFKRADIYSVGLVYWEIARRCSVGGIVEEYQLPYYD
                *******************:.****************************:**********

mouse_alk7      MVPSDPSIEEMRKVVCDQKLRPNLPNQWQSCEALRVMGRIMRECWYANGAARLTALRVKK
rat_alk7        MVPSDPSIEEMRKVVCDQKLRPNLPNQWQSCEALRVMGRIMRECWYANGAARLTALRVKK
human_alk7      MVPSDPSIEEMRKVVCDQKFRPSIPNQWQSCEALRVMGRIMRECWYANGAARLTALRIKK
                *******************:**.:*********************************:**

mouse_alk7      TISQLCVKEDCKA
rat_alk7        TISQLCVKEDCKA
human_alk7      TISQLCVKEDCKA
```

N150 → H
I195 → T
I482 → V

Figure S2. Alignment of ALK7 human and mouse protein sequences

Acvr1c variants are color labeled as indicated. *, : and . denote identical, conserved, and non-conserved amino acid residues, respectively.
