# Supplementary material for: Human ACVR1C missense variants that correlate with altered body fat distribution produce metabolic alterations of graded severity in knock-in mutant mice
Source: Mol Metab. 2024 Feb 1;81:101890. doi: 10.1016/j.molmet.2024.101890 (PMC10863331; doi:10.1016/j.molmet.2024.101890)
Supplement: Multimedia component 4 [file mmc4.pdf]

```
mouse_ALK4      MAESAGASSFFPLVVLLLAGSGGSGPRGIQALLCACTSCLQTNYTCETDGACMVSIFNLD
mouse_ALK5      -MEAAAAAPRRPQLLIVLVAAATLLP-GAKALQCFCHLCTKDNFTCETDGLCFVSVTETT
mouse_ALK7      MTPARGSALSLALLLVALAAD------LAAGLKCVCLLCDSSNFTCQTEGACWASVMLTN
                   : .::   . ::: *..          .* * *  * . *:**:*:* * .*:

mouse_ALK4      GVEHHVRTCIPKVELVPAGKPFYCLSSE---DLRNTHCCYIDFCNKIDLRVPSGHLKEPA
mouse_ALK5      DKVIHNSMCIAEIDLIPRDRPFVCAPSSKTGAVTTTYCCNQDHCNKIELPT-TGPFSE-K
mouse_ALK7      GKEQVIKSCVSLPELN---AQVFCHSSN---NVTKTECCFTDFCNNITLHLPTASPNA--
                .       *:.  :*      . * .*.    : .* **  *.**:* *   :.  .

mouse_ALK4      HPSMWGPVELVGIIAGPVFLLFLIIIIVFLVINYHQR--VYHNRQRLDMEDPSCEM-CLS
mouse_ALK5      QSAGLGPVELAAVIAGPVC--FVCIALMLMVYICHNR--TVIHHRVPNEEDPSLDRPFIS
mouse_ALK7      --PRLGPTELTVVITVPVC--LLSIAAMLTIWACQDRQCTYRKTKRHNVEEALAEYSLVN
                  .  **.**. :*: **   :: *  :: :   ::*  .  : :  : *:.  :   :.

mouse_ALK4      KDKTLQDLVYDLSTSGSGSGLPLFVQRTVARTIVLQEIIGKGRFGEVWRGRWRGGDVAVK
mouse_ALK5      EGTTLKDLIYDMTTSGSGSGLPLLVQRTIARTIVLQESIGKGRFGEVWRGKWRGEEVAVK
mouse_ALK7      AGKTLKDLIYDATASGSGSGLPLLVQRTIARTIVLQEIVGKGRFGEVWHGRWCGEDVAVK
                 ..**:**:** ::*********:****:******** :*********:*:* * :****

mouse_ALK4      IFSSREERSWFREAEIYQTVMLRHENILGFIAADNKDNGTWTQLWLVSDYHEHGSLFDYL
mouse_ALK5      IFSSREERSWFREAEIYQTVMLRHENILGFIAADNKDNGTWTQLWLVSDYHEHGSLFDYL
mouse_ALK7      IFSSRDERSWFREAEIYQTVMLRHENILGFIAADNKDNGTWTQLWLVSEYHEQGSLYDYL
                *****:******************************************:***:***:***

mouse_ALK4      NRYTVTIEGMIKLALSAASGLAHLHMEIVGTQGKPGIAHRDLKSKNILVKKNGMCAIADL
mouse_ALK5      NRYTVTVEGMIKLALSTASGLAHLHMEIVGTQGKPAIAHRDLKSKNILVKKNGTCCIADL
mouse_ALK7      NRNIVTVAGMVKLALSIASGLAHLHMEIVGTQGKPAIAHRDIKSKNILVKKCDTCAIADL
                **  **: **:***** ******************.*****:********* . *.****

mouse_ALK4      GLAVRHDAVTDTIDIAPNQRVGTKRYMAPEVLDETINMKHFDSFKCADIYALGLVYWEIA
mouse_ALK5      GLAVRHDSATDTIDIAPNHRVGTKRYMAPEVLDDSINMKHFESFKRADIYAMGLVFWEIA
mouse_ALK7      GLAVKHDSIMNTIDIPQNPKVGTKRYMAPEMLDDTMNLSIFESFKRADIYSVGLVYWEIA
                ****:**:  :****. * :**********:**:::*:. *:*** ****::***:****

mouse_ALK4      RRCNSGGVHEDYQLPYYDLVPSDPSIEEMRKVVCDQKLRPNVPNWWQSYEALRVMGKMMR
mouse_ALK5      RRCSIGGIHEDYQLPYYDLVPSDPSVEEMRKVVCEQKLRPNIPNRWQSCEALRVMAKIMR
mouse_ALK7      RRCSVGGVVEEYQLPYYDMVPSDPSIEEMRKVVCDQKLRPNLPNQWQSCEALRVMGRIMR
                ***. **: *:*******:******:********:******:** *** ******.::**

mouse_ALK4      ECWYANGAARLTALRIKKTLSQLSVQEDVKI
mouse_ALK5      ECWYANGAARLTALRIKKTLSQLSQQEGIKM
mouse_ALK7      ECWYANGAARLTALRVKKTISQLCVKEDCKA
                ***************:***:***. :*. *
```

Transmembrnae domains

ALK7 Ile^{195} (ALK5 I^{205})

ALK7 Ile^{482} (ALK5 Leu^{492})

ALK7 Asn^{150} (ALK5 Asn^{160})

FKBP12 binding site in ALK5: Leu^{193}, Pro^{194}, Leu^{195}, Leu^{196}, Gln^{198}, Trp^{242}, Phe^{243}

Hydrophobic environment of ALK5 Ile^{205}: Leu^{207}, Val^{229}, Val^{239}, Trp^{277}, Val^{279}

Hydrophobic environment of ALK5 Ile^{492}: Ile^{303}, Phe^{408}, Leu^{495}

Figure S3. Alignment of mouse ALK4, ALK5 and ALK7 protein sequences. Shown are the locations of ALK7 missense variants and structurally or functionally important residues in ALK5 kinase domain as derived from Huse et al. 1999.
