# Supplementary material for: Human ACVR1C missense variants that correlate with altered body fat distribution produce metabolic alterations of graded severity in knock-in mutant mice
Source: Mol Metab. 2024 Feb 1;81:101890. doi: 10.1016/j.molmet.2024.101890 (PMC10863331; doi:10.1016/j.molmet.2024.101890)
Supplement: Multimedia component 5 [file mmc5.pdf]

Figure S4

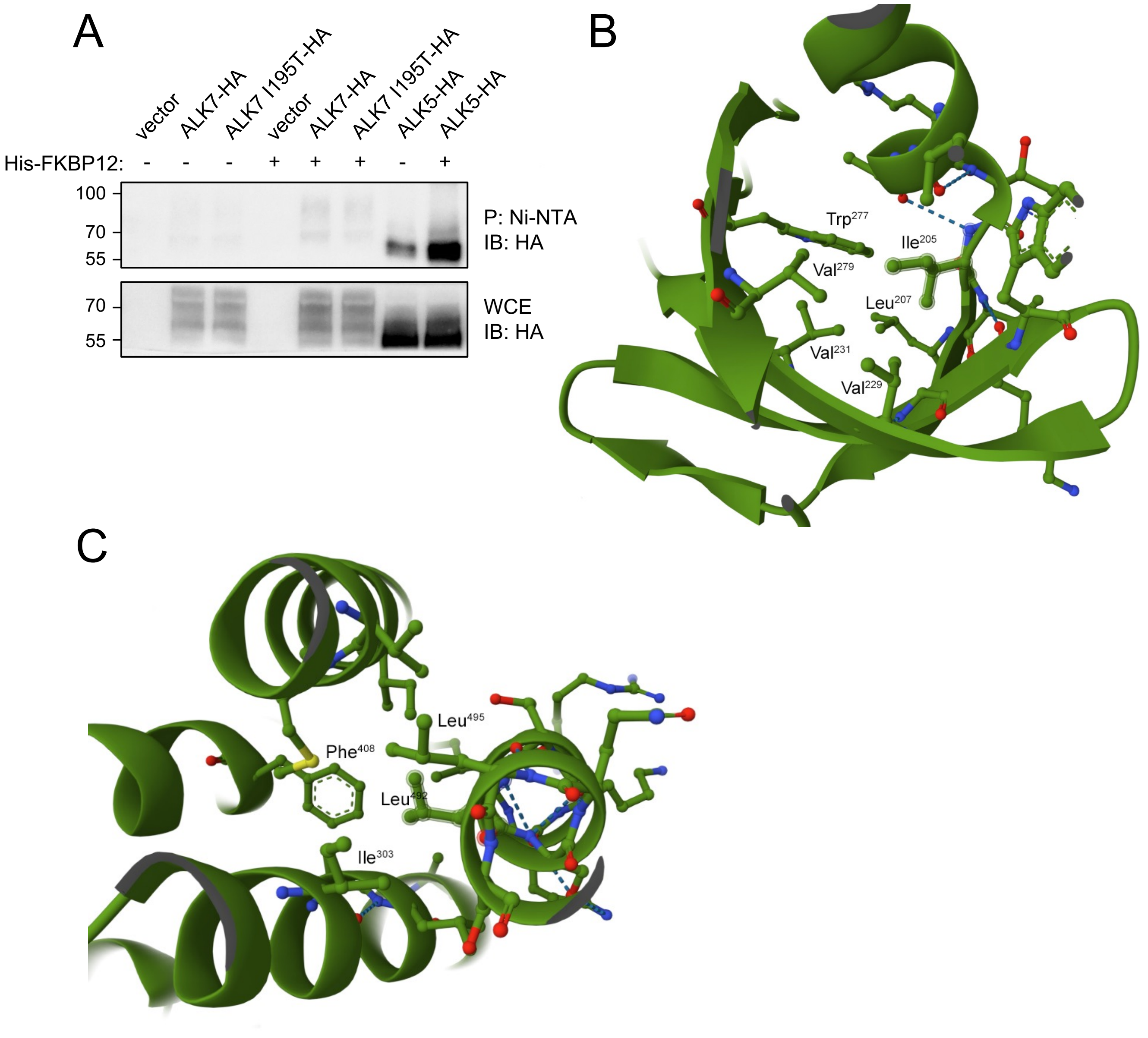

Figure S4. Assessment of possible functional and structural importance of ALK7 residues Ile^{195} and Ile^{482}

(A) Assessment of interaction between FKBP12 and ALK7, ALK7-I195T and ALK5 by precipitation and Western blotting in transfected HEK cells. His-tagged FKBP12 was precipitated (P) with Ni-NTA beads and blots probed (IB) with antibodies against HA-tagged ALK5 and ALK7. WCE: whole cell extract.

(B) Structural environment of ALK5 Ile^{205} (homologous to ALK7 Ile^{195}) in the ALK5 kinase domain (derived from PDB: 1b6c; Huse et al. 1999).

(C) Structural environment of ALK5 Leu^{492} (homologous to ALK7 Ile^{482}) in the ALK5 kinase domain (derived from PDB: 1b6c; Huse et al. 1999).
